# Expression of the Human Serotonin 5-HT_7_ Receptor Rescues Phenotype Profile and Restores Dysregulated Biomarkers in a *Drosophila melanogaster* Glioma Model

**DOI:** 10.3390/cells11081281

**Published:** 2022-04-09

**Authors:** Florestan Courant, Marion Maravat, Wanyin Chen, David Gosset, Lauren Blot, Nadège Hervouet-Coste, Vincent Sarou-Kanian, Séverine Morisset-Lopez, Martine Decoville

**Affiliations:** 1 Centre de Biophysique Moléculaire—CBM, UPR 4301, CNRS, Rue Charles Sadron, CEDEX 02, F-45071 Orléans, France; florestan.courant@inserm.fr (F.C.); wanyin.chen@cnrs-orleans.fr (W.C.); david.gosset@cnrs.fr (D.G.); lauren.blot@laposte.net (L.B.); nadege.hervouet@cnrs-orleans.fr (N.H.-C.); martine.decoville@cnrs-orleans.fr (M.D.); 2 Conditions Extrêmes et Matériaux: Haute Température et Irradiation—CEMHTI-CNRS UPR 3079, CEDEX 02, F-45071 Orléans, France; m.maravat@gmail.com (M.M.); vincent.sarou-kanian@cnrs-orleans.fr (V.S.-K.); 3 UFR Sciences et Techniques, Université d’Orléans, 6 Avenue du Parc Floral, F-45100 Orléans, France

**Keywords:** GPCR, serotonin, 5-HT_7_ receptor, glioma, Drosophila, HR-MAS NMR, epigenetics

## Abstract

Gliomas are the most common primary brain tumors in adults. Significant progress has been made in recent years in identifying the molecular alterations involved in gliomas. Among them, an amplification/overexpression of the EGFR (Epidermal Growth Factor Receptor) proto-oncogene and its associated signaling pathways have been widely described. However, current treatments remain ineffective for glioblastomas, the most severe forms. Thus, the identification of other pharmacological targets could open new therapeutic avenues. We used a glioma model in *Drosophila melanogaster* that results from the overexpression of constitutively active forms of EGFR and PI3K specifically in glial cells. We observed hyperproliferation of glial cells that leads to an increase in brain size and lethality at the third instar larval stage. After expression of the human serotonin 5-HT_7_ receptor in this glioma model, we observed a decrease in larval lethality associated with the presence of surviving adults and a return to a normal morphology of brain for some Drosophila. Those phenotypic changes are accompanied by the normalization of certain metabolic biomarkers measured by High-Resolution Magic Angle Spinning NMR (HR-MAS NMR). The 5-HT_7_R expression in glioma also restores some epigenetic modifications and characteristic markers of the signaling pathways associated with tumor growth. This study demonstrates the role of the serotonin 5-HT_7_ receptor as a tumor suppressor gene which is in agreement with transcriptomic analysis obtained on human glioblastomas.

## 1. Introduction

Gliomas are the most common brain tumors representing almost 50% of brain cancers. Of great heterogeneity, the management of these tumors is difficult, and the morbidity rate of patients is high, regardless of the degree of malignancy of these tumors. Gliomas are classified into four categories according to their degree of malignancy, from grade 1, the most benign, to grade 4, glioblastomas (GBMs), which are among the most frequent and most aggressive brain tumors in humans. Recently, the World Health Organization (WHO) has refined the classification of gliomas by considering additional phenotypic and genetic parameters [1].

Many genetic alterations causing gliomas have been identified. The most common found in GBM corresponds to mutation and/or overexpression of tyrosine kinase (RTK) receptors such as the Epidermal Growth Factor Receptor (EGFR) receptor. Alterations in the RAS/MAPK pathway and the phosphatidylinositide 3 kinase (PI3K)-AKT-mTOR pathway which regulate cell proliferation and apoptosis have also been identified. In addition, mutations in genes encoding isocitrate dehydrogenases 1 and 2 (IDH1 and IDH2) are also found in low grade gliomas (grades II and III) and secondary GBMs (GBM, IDH-mutant) [1].

Depending on their severity, treatment of GBMs consists of surgical removal, possibly combined with chemotherapy and/or radiotherapy. If in the 1990s treatments were based on the use of cytotoxic drugs [2], more recently patients benefit from molecular targeted therapy which involved in inhibiting receptor tyrosine kinases such as EGFR, PDGFR (Platelet-Derived Growth Factor Receptor) or VEGFR (Vascular Endothelial Growth Factor Receptor), as well as kinases in signaling pathways such as the PI3K pathway [3]. Thus, while the use of EGFR phosphorylation inhibitors has raised hope, clinical trials with anti-EGFR drugs have been disappointing [4]. Tumors become resistant to treatment and use strategies to bypass the inhibited pathways. This resistance is particularly due to the existence of a tumor niche inhabited by cancer stem cells that would be able to maintain their undifferentiated state and their capacity for self-renewal, which would condition tumor progression. New avenues are being explored that target metabolic pathways and epigenetic processes. Several studies have shown epigenetic abnormalities of glioma cells [5,6,7] such as aberrant DNA methylation [8,9], abnormal microRNA (miRNA) [10], chromatin remodeling [11] and histone modifications [12,13]. Inhibition of histone deacetylases (HDACs) and histone methyltransferases may be new approaches to treat gliomas [7,14]. For example, inhibitors of HDACs have been used in combination with radiotherapy and chemotherapy [15,16]. Inhibitors of G9a, a histone methyltransferase, have also been shown to be potential new drugs for the treatment of gliomas [13,17]. However, given the complexity of glioma development, application of such therapies remains limited.

As many other cancer cells, glioma cells modify their metabolic pathways to produce energy and to proliferate. This abnormal metabolism may be related to alterations of RTKs and IDH mutations, such as the RTK/PI3K/AKT/mTOR pathways which regulates glucose and glutamine metabolism. Thus, targeting these metabolic related pathways would be of great interest in therapeutics [18,19]. For example, glioma cells use the Warburg effect (aerobic glycolysis) to produce energy [20,21]. They increase glucose intake and its product pyruvate is converted to lactate through fermentation. Several studies have shown that negative regulators of the Warburg effect suppresses tumor growth [22,23].

Serotonin (5-HT) has recently emerged as a growth factor on several types of tumor cells. It was shown to stimulate proliferation, migration and invasion of glioma cells in vitro and it was thus functionally related to oncogenesis [24]. However, the effects of serotonin on tumor growth are still unclear since opposing effects have been described [25,26]. Some studies reported an inhibitory effect of serotonin on tumor growth, mainly via vasoconstrictive effects on tumor vessels [27]. These studies suggest that the effect of serotonin is concentration dependent; high doses having a mitogenic effect on tumor cells, whereas low doses reduce tumor growth. In addition, this differential effect may be related to the interaction of serotonin with a variety of 5-HT cell surface receptors. Fourteen subtypes of 5-HT receptors (5-HTR) that mediate the actions of 5-HT have been cloned [28]. Based on the sequence homology and transduction to second messenger, they are divided into seven different subclasses: 5-HT_1A-F_, 5-HT_2A-C_, 5-HT_3_, 5-HT_4_, 5-HT_5A-B_, 5-HT_6_ and 5-HT_7_ receptors. With the exception of the 5-HT_3_R receptor, which is an anion channel, 5-HT receptors are G protein-coupled receptors (GPCR). Among them, we investigated the role of 5-HT_7_ receptor (5-HT_7_R) on gliomagenesis. The 5-HT_7_R is the last serotonin receptor subtype to be discovered in 1993 [29,30,31]. It is a G protein coupled receptor, highly expressed in two compartments including the central nervous system (CNS) [32] and the gastrointestinal tract [33], but it is also found in other tissues such as the immune cells [34]. In agreement with this tissue distribution, the receptor was shown to regulate important pathophysiological processes and has become a promising target in the treatment of CNS disorders such as sleep disorders, migraine, neuropathic pain or neuro-psychiatric disorders [35,36,37] as well as inflammatory and immune-mediated disorders [34]. The 5-HT_7_R is coupled to Gs protein, resulting in an increase in intracellular cAMP in cells. This signaling induces AKT and ERK activation through PKA activation and RAS protein [38]. It has been also demonstrated that the ERK1/2 activation pathway involved cAMP-induced activation of the nucleotide exchange factor (GE) such as Epac 1 and Epac2 [38]. The 5-HT_7_R interacts with the G12, a member of the G protein family which can activate downstream effectors of the Rho family of small GTPases (Rho, Rac and Cdc42) [39]. Interestingly, several arguments led us to speculate and investigate its role in glioma. First, it was found that eight human glioblastoma cell lines tested, U-373 MG, U-138MG, U-87MG, DBTRG-05MG, T98G, H4, CCF-STTG1 and Hs 683 expressed functioning 5-HT_7_R [40]. Secondly, on the U-373MG glioblastoma cell line, its activation induces Interleukin6 production which is also involved in glioma proliferation [41]. Third, stimulation of 5-HT_7_R induces extracellular regulated kinase 1/2 (ERK1/2) phosphorylation, a well-known pathway involved in migration and proliferation of glioma [42] and related to EGF receptor expression [43]. Finally, the role of the receptor in other aggressive cancers, such as triple-negative breast cancer [44], hepatocellular carcinoma [45] underlies its role in tumor growth in vivo.

Currently, the development of new anti-cancer agents is a long and costly process, largely due to the failure of some drug candidates to be effective in vivo, even though they showed promising activities in initial in vitro tests. Thus, it appears important to identify new therapeutic targets for the treatment of these highly resistant cancers in more integrative models. Most models to evaluate gliomagenesis are performed in rodents, and in particular in mice, which have been obtained by transplanting glioma cells into the brain or by using transgenic mice developing gliomas. While these models have a number of advantages, they also have several limitations: the use of particular lines often used (immunodeficient), the duration of the experiment, the high cost and ethical considerations. *Drosophila melanogaster* can be considered as an interesting alternative model organism which has been used for many years to study complex biological processes. Indeed, it has multiple advantages: (1) the rearing conditions are simple, (2) the development cycle is rapid from 10 to 15 days, (3) it is very prolific, and (4) many mutants are available [46]. Moreover, in the last few decades, very sophisticated genetic tools have been developed in *Drosophila melanogaster* (germline transformation, control of gene expression in space and time, mitotic recombination) [47]. The comparison of its genome with that of humans has shown that 70% of the genes involved in human diseases have an equivalent in *Drosophila melanogaster* [48]. In addition, a large number of signaling pathways are highly conserved between Drosophila and humans; in particular, signaling pathways involving receptor tyrosine kinases. *Drosophila melanogaster* is also a very practical model for pharmacological studies, as it can be easily administered molecules of interest either by injection, food or inhalation [49,50,51,52]. The Drosophila nervous system shows amazing conservation in its cellular composition and developmental mechanisms [53]. In particular, *Drosophila melanogaster* has several types of glial cells according to their functions and gene expression [54]. *Drosophila melanogaster* thus appears to be a very good model in cancer research [55] and, in particular, in brain cancer [56,57,58]. In addition, the presence of a large diversity of GPCR genes have been identified in *Drosophila melanogaster*. Among the 100 genes that encode putative neurotransmitter and hormone GPCR receptors, three encode serotonergic receptors, i.e., the 5-HT_1_A/BDro [59], 5-HT_2_Dro [60] and 5-HT_7_Dro [61]. Interestingly, they are associated with the same signaling pathways and the same type of behavioural responses as those described in mammals [62,63].

In this study, we aim at investigating the role of 5-HT_7_ receptor on glioma progression in *Drosophila melanogaster*. We used the glioma model established by Read et al. [55]; This model is obtained by co-expressing constitutive forms of PI3K and EGFR in glial cells throughout development. Abnormal glial proliferation similar to human glioma is observed leading to deformed and larger brains, and to lethality during the third instar larval stage. We have explored the effect of 5-HT_7_ receptor on glioma development by using characteristic markers of the signaling pathways associated with tumor growth as well as epigenetic and metabolic biomarkers modifications.

## 2. Materials and Methods

### 2.1. TCGA

The mRNA expression, clinical data were downloaded from The Cancer Genome Atlas (TCGA http://cancergenome.nih.gov, 13 March 2022) using the GlioVis data portal for visualization and analysis of brain tumor expression datasets [64]. For mRNA analysis, RNA-seq has been performed. The normalized count reads from the pre-processed data (sequence alignment and transcript abundance estimation) were log2 transformed after adding a 0.5 pseudocount (to avoid infinite value upon log transformation).

### 2.2. Transgenic Lines

For 5-HT_7_R expression studies, *UAS-5-HT_7_R* constructs were obtained by cloning the full-length *Drosophila d5-HT_7_R* cDNA from LD04507 obtained from the Drosophila genomic Resource Center and the cDNA encoding isoform A of the human h5-HT_7_R (plasmid obtained from the cDNA resource center, www.cdna.org, 13 March 2022) in the pTFW transformation vector, allowing expression of a tagged protein (3×FLAG at the N-terminus). Transgenic lines were obtained by BestGene Inc. Service (Chino Hills, CA, USA).

### 2.3. Drosophila Culture and Genetics

The fly stocks were maintained at 22 °C on a standard medium (per liter: 90.25 g of cornmeal, 82.5 g of dry yeast, 10.75 g of agar and 37.5 mL of a 10% solution of methyl-4-hydroxybenzoate in ethanol). Crosses were performed at 26 °C on standard medium except when indicated. Drosophila lines were obtained from the Bloomington Stock Center except the stock *UAS-dEGFR^λ^* (T. Schubach).

To express 5-HT_7_R in the dorsal wing compartment, females *Bx-Gal4* were crossed with *UAS-5-HT_7_R* males. Expression in the posterior compartment of the wing was obtained by crossing with *en-Gal4/Cy* flies. *UAS-GFP; repo-Gal4/TMTbSb* flies were crossed with *UAS-5-HT_7_R* for glial cells expression. Glioma were generated by crossing *UAS-dp110^CAAX^; UAS-dEGFR^λ^* virgin females with *UAS-GFP; repo-Gal4/TMTbSb* males. The effect of h5-HT_7_R expression on glioma was investigated by crossing *UAS-dp110^CAAX^; UAS-h5-HT_7_R; UAS-dEGFR^λ^* virgin females with *UAS-GFP; repo-Gal4/TMTbSb* males. Gal4 controls (*repo>GFP*) were obtained by crossing *w^1118^* virgins with *UAS-GFP; repo-Gal4/TMTbSb. UAS* controls were obtained by crossing *w^1118^* males with *UAS-5-HT_7_R*, or *UAS-dp110^CAAX^; UAS-dEGFR^λ^* or *UAS-dp110^CAAX^; UAS-h5-HT_7_R; UAS-dEGFR^λ^* virgins (UAS controls). Larval brains were recovered from third instar wandering larvae (120 h after egg deposition).

### 2.4. Lifespan Analysis

Adult flies emerging within a 24-h period were collected under CO_2_ anesthesia. Groups of less than 25 flies were placed in vials and incubated at 26 °C. They were transferred into fresh vials every 2 or 3 days, and the number of surviving flies was recorded daily. Data correspond to the percentage of surviving flies as a function of time.

### 2.5. cAMP Accumulation and Functional Assays

We used the LANCE^®^ Ultra cAMP assay (Perkin-Elmer Life Sciences, Boston, MA, USA), a homogeneous time-resolved fluorescence resonance energy transfer (TR-FRET) immunoassay, to measure cAMP produced by cells in Drosophila brains. A cAMP standard curve allowed us to determine assay sensitivity (IC50 value) and dynamic range (IC10–IC90). It also provided a means to translate the measured TR-FRET signal into actual quantities of cAMP produced in brain lysates. We diluted brain lysates in Hank’s balanced salt solution (HBSS) containing HEPES pH 7.4, 1 mM isobutylmethylxanthine (IBMX), 1 mg/mL BSA. Then, 10 µL of each sample cAMP standard serial dilutions (from 10^−11^ to 10^−6^ M) and brain lysates in triplicate wells, were dispensed in white 384-well microtiter plates (Optiplate, Perkin Elmer, Boston, MA, USA). Then, the ULight-anti-cAMP antibody solution (5 µL) and the Eu-cAMP tracer have been added to each well. After 2 h of incubation at room temperature in the dark, the plates were read on a VictorV microplate reader (Perkin-Elmer Life Sciences, Boston, MA, USA). Free cAMP produced in Drosophila brains competes with the Eu-cAMP tracer for the binding to the ULight-mAb, causing a decrease in TR-FRET signal. Counts at 665 nm obtained in cAMP standard curves allow interpolating the amount of cAMP produced in brains.

### 2.6. Western Blots

Brains from third instar larvae were crushed in cold PBS containing protease inhibitors (Roche Complete^™^ Protease Inhibitor Cocktail) and phosphatase inhibitors (Roche Phosstop) and then heated at 95 °C for 7 min. Samples were loaded in 8% or 15% SDS-PAGE and transferred onto nitrocellulose membranes (Bio-Rad). Then, membranes were blocked for one hour at room temperature in 5% BSA or 5% skim milk in TBS-0.1% Tween20 (TBS-T) according to the manufacturer’s instructions. Mouse antibodies against phospo-p70 S6K^Thr389^ (#9206), rabbit antibodies against phospho-AMPKα^Thr172^ (#2535), AMPKα (#2532), p70 S6K (#9202).phospho--4E-BP1^Thr37/46^ (#2855), non-phosphor-4E-BP1 (#4923) were purchased from Cell signaling. GFP and α-tubulin were used as loading controls. Mouse antibodies against α-tubulin (sc-8035) were purchased from Santa Cruz Biotechnology and mouse monoclonal antibodies against YFP (ABIN1545635) were obtained from antibodies-online GmbH. Blots were incubated with primary antibodies diluted in 5% BSA or 5% skim milk in TBS-T overnight at 4 °C. After three washes with TBS-T, the blots were probed with secondary antibody for one hour at room temperature. Goat anti-Rabbit IgG (H+L) Cross-Adsorbed Secondary Antibody, HRP (#65-6120) and Rabbit anti-Mouse IgG (H+L) Cross-Adsorbed Secondary Antibody, HRP (#816720) were purchased from Invitrogen. Protein bands were detected with ClarityMax^TM^ Western ECL Blotting Substrate (#1705062) by using ChemiDoc^TM^ Imaging systems from BIO-RAD.

The protein bands were quantified with ImageLab software (BIO-RAD). The measured values of phosphorylated protein level and total protein were first normalized with loading controls, then the ratios of phosphorylated protein to total protein were normalized with control fly’s sample and shown as fold change in phosphorylation. The mean-fold change of independent Western blots were analyzed with Prism and the significance was calculated by Kruskal–Wallis test (one-way ANOVA, Kruskal–Wallis test, Dunn’s multiple comparison test, n = 6).

### 2.7. Flow Cytometry

The samples were prepared following Harzer et al. [65]. Brains from control larvae (*repo*>*GFP*), glioma model (*repo*>*UAS-dp110^CAAX^; UAS-dEGFR^λ^*) or h5-HT_7_R glioma model (*repo*>*UAS-dp110^CAAX^*; *UAS-h5-HT_7_R; UAS-dEGFR^λ^*) were dissected under binocular magnifier in Schneider medium (Gibco 2170-024) with 10% SVF (SVF Sigma F7524) and collected in 1.5 mL of 1× Rinaldini buffer (8 g/L of NaCL, 0.2 g/L of KCL, 44 mg/L of NAH_2_PO_4_, 1 g/L of NaHCO_3_, 1 g/L of glucose, 0.88 g/L of Na_3_C_6_H_5_O_7_). After two washings in 1× Rinaldini buffer, the brains were incubated with dissociation buffer (Schneider medium with 20 µg/mL of collagenase) for 1 h at 30 °C with frequent gentle shaking. Then, the brains were carefully washed two times with 1× Rinaldini buffer and two times with Schneider SVF medium. The brains were then dissociated in 200 µL of Schneider SVF medium by pipetting up and down 100 times. The mixture was 30 µM filtered and centrifuged at 300 g for 13 min.

Cell pellets were fixed in 4% PBS-PFA for 15 min and then washed two times with 1× PBS. Cells were permeabilized by incubating them for 45 min in permeabilization/blocking solution (1× PBS, 3% BSA and 0.02% Triton X-100) in the dark at room temperature. After centrifugation, pellet was divided in 3 in the same buffer (for white cells, isotypes and protein labelling) and incubated with antibodies for 1 hour at 4 °C in dark (list of antibodies used is presented in Table S1). Then, cells were pelleted, and the supernatant was discarded. 1× PBS with 3% BSA was added onto the pellet without disturbing it and samples were stocked at 4 °C in the dark over 2 days.

Glioma cells were analyzed on Fortessa X20 Becton Dickinson flow cytometer. Glial cells were selected by 488 nm excitation and detection at 530 nm, band pass 30 nm, to GFP expression. Epigenetic modifications were analyzed by 633 nm excitation, detection at 670 nm, band pass 30 nm, for Alexa fluor 647 (APC) and 405 nm excitation, detection 450 nm, band pass 50 nm for Pacific Blue (BV421). Control profiles (white cells and isotypic controls) are shown on Figure S1.

### 2.8. HR-MAS NMR-Based Metabolic Analysis

Brain samples for HR-MAS NMR spectroscopy were prepared as described previously [66]. Each sample was inserted into a 4 mm zirconia rotor and was analyzed on a Bruker 750 MHz Avance III HD spectrometer (Bruker BioSpin, Karlsruhe, Germany) equipped with a Bruker 4 mm MAS probe. The temperature in the rotor was kept at 3 °C during the duration of the experiment and the rotor was spun at a frequency of 4 kHz. 1D ^1^H HR-MAS NMR data were acquired using a spin echo pulse program (an echo time of 2 rotor periods) with water presaturation (1-second pulse duration with low power) and the following parameters: spectral width: 13 ppm; number of scans: 1024; and recycling delay: 2 s for a total acquisition time of 53 min. Prior to the Fourier Transform, the Free Induction Decay (FID) was multiplied by an exponential line broadening of 0.3 Hz. Supplementary 2D shows ^1^H-^1^H total through bond correlation spectroscopy (TOBSY) acquisition was performed on a few samples for metabolite identification purposes.

For data processing, NMR raw data was phased using TopSpin 3.5 then pre-processed using NMRProcFlow [67]. The pre-processing included baseline correction, spectral alignment and deletion of the water region (5.15 to 4.7 ppm). Then, the spectra were reduced by 0.01 ppm buckets in order to obtain a data matrix which was imported in MetaboAnalyst 5.0 to be normalized by total sum and auto scaled [68].

All the statistical analyses were done with MetaboAnalyst 5.0 [68]. Principal Component Analysis (PCA) was first performed to detect any clustering of samples and the presence of outliers. Then, Partial Least Square Discriminant Analysis was performed to build a prediction model on our data set using a standard method scaling and CV cross-validation method. Model validation was done by permutation testing based on separation distance using 100 iterations. The predictive ability of the model is assessed by Q2 which is obtained during cross-validation. To be considered highly predictive, the model had to have a Q2 greater or close to 0.8.

To visualize the variations in relative levels of the first 25 significantly altered buckets, heat maps were constructed on group averages. Euclidian distance with Ward’s clustering method was used based on one-way ANOVA (*p* < 0.05).

Metabolite identification was achieved using Chenomx NMR suite 8.6.

## 3. Results

### 3.1. 5-HT_7_R Expression Is Dysregulated in Human GBM

**Figure 1:**
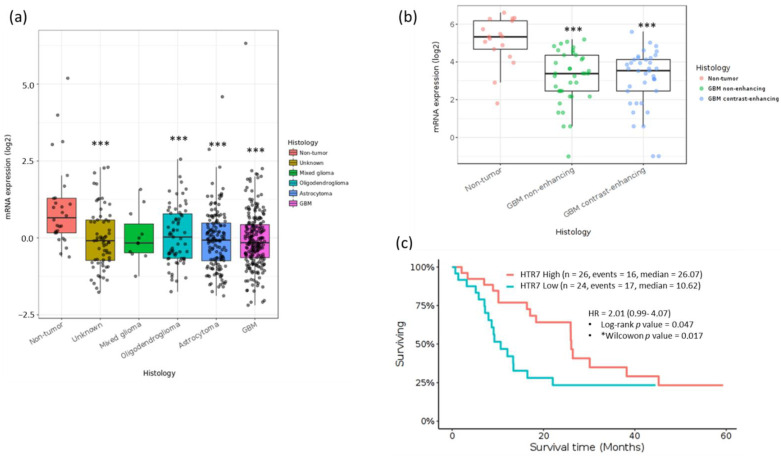
5-HT_7_R mRNA expression in human brain cancers. (**a**) 5-HT_7_R is lower expressed in many brain tumors including GBM compared with normal tissues, data from Rembrandt. (**b**) The mRNA expression of the 5-HT_7_R was lower in tumor tissues of GBM patients than in the corresponding normal tissues, data from Gill database) *** *p* < 0.001, Tukey’s Honest Significant Difference (HSD); (**c**) Kaplan–Meier estimator survival analysis from Nutt datasets. * *p* < 0.05, Wilcoxon-Mann-Whitney test. The split in two groups, HTR7 low and HTR7 high has been performed by the median. Data from TCGA, http://cancergenome.nih.gov, 13 March 2022.

In order to explore whether 5-HT_7_R expression is dysregulated in GBM patients, we analyzed data from TCGA by using GlioVis data porta. The mRNA expression of the 5-HT_7_R has been evaluated in various types of human brain cancer, in oligodendroglioma (67 samples), astrocytoma (147 samples) and GBM (219 samples) from Rambrandt database. A significant decrease in 5-HT_7_R expression has been observed in all types of tumors compared to the control samples (28 samples) (Figure 1a). In addition, analysis of enhancing and non-enhancing parts of glioblastoma (37 samples and 38 samples, respectively, from Gill database) shows a significant decrease in 5-HT_7_R expression in both tumor samples compared to controls (17 samples) (Figure 1b). In addition, Kaplan–Meier curves show a significant difference in overall survival between patients with high and low-5-HT_7_R expression in GBM in TCGA-Nutt database (Figure 1c). Therefore, low expression of 5-HT_7_R is correlated with a poor prognostic of patient survival.

In agreement with a previous study showing expression of 5-HT_7_R in various human glioma cell lines [40], these results underlie the interest of the 5-HT_7_R in the context of glioma. In order to go further and explore its role on cell proliferation and tumor growth, we expressed the receptor in vivo in *Drosophila melanogaster*.

### 3.2. Effect of 5-HT_7_R Expression in Drosophila

**Figure 2:**
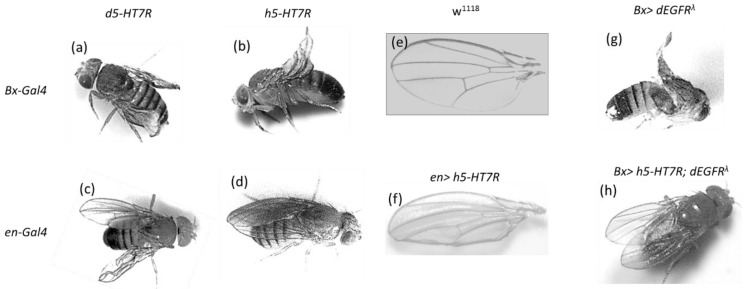
Drosophila or human 5-HT_7_ receptor expression affects wing patterning. (**a**,**c**) Expression of the Drosophila d5-HT_7_R, (**b**,**d**) expression of the human h5-HT_7_R. Expression in the dorsal compartment (Bx-Gal4) induces “curly” wings (**a**,**b**). Expression in the posterior compartment (en-Gal4) mimics “rolled” phenotypes (**c**,**d**). (**e**) Wing from wild-type control (w^1118^), (**f**) wing from (**d**). Expression of a constitutive form of Drosophila EGFR (dEGFR^λ^) alters wing patterning (**g**). Expression of h5-HT_7_R suppresses the phenotype induced by dEGFR^λ^ (**h**).

First, we wanted to assess the effect of 5-HT_7_ receptor expression in a non-nervous tissue. For this, we have chosen the Drosophila wing which is not essential for Drosophila viability. We used two Gal4 drivers: Bx-Gal4 and en-Gal4 which express Gal4, respectively, in the dorsal and posterior compartment of the wing disc. Several independent transgenic lines expressing 5-HT_7_R were used and all exhibit the same phenotypes. Here, we present results from lines with insertion on the second chromosome. Interestingly, expression of Drosophila (d5-HT_7_R) or human 5-HT_7_R (h5-HT_7_R) in the wing imaginal disc affects wing patterning (Figure 2). When expressing d5-HT_7_R in the dorsal compartment, wing patterning is severely affected, and we observed a “curly-like” phenotype (Figure 2a). Expression of h5-HT_7_R results in a milder phenotype with some F1 progeny also showing a “curly”-like phenotype (Figure 2b). Such an observation indicates that the dorsal compartment is smaller than the ventral one and hence suggests that cell proliferation has been reduced in the dorsal compartment.

Expression of d5-HT_7_R in the posterior compartment of the wing disc leads to abnormal wing blade development (Figure 2c). The phenotype observed after expression of h5-HT_7_R is weaker, with the posterior wing edge “rolled” (Figure 2d,f). These phenotypes are reminiscent of hypomorphic alleles of rolled (dEGFR). We then investigated whether the expression of h5-HT_7_R could suppress the effect of a dEGFR gain-of-function allele (dEGFR^λ^ which encodes a constitutive form of dEGFR). Expression of dEGFR^λ^ in the dorsal compartment of the wing (Bx driver) strongly affects wing patterning (Figure 2g). When co-expressing dEGFR^λ^ and h5-HT_7_R, we observed some F1 showing an almost rescued phenotype (Figure 2h).

**Figure 3:**
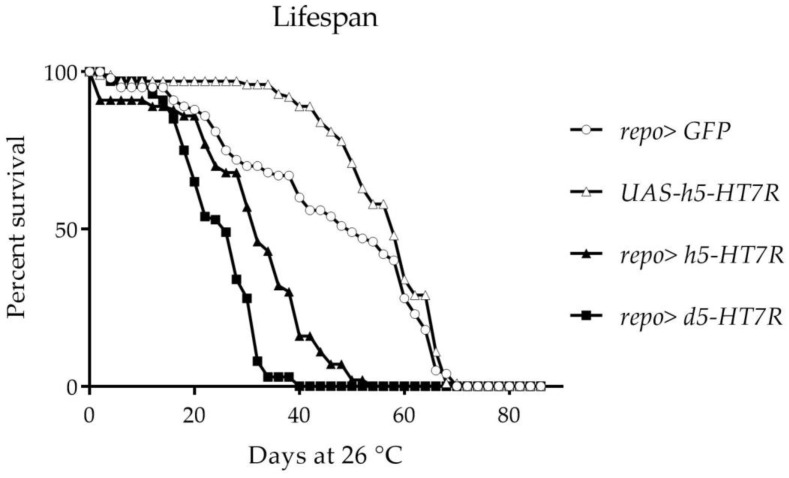
Expression of Drosophila or human 5-HT_7_ receptor in glial cells reduces lifespan of flies. Controls are *repo>GFP* and UAS-h5-HT_7_R. Results correspond to the percent of living flies.

All these results suggest that expression of the Drosophila or human 5-HT_7_R in the wing imaginal disc reduces cellular proliferation and may interfere with EGFR signaling. We then wanted to ensure that expression in glial cells during development is not lethal and does not significantly affect the development of the larval brain. For that purpose, we used the Gal4-repo driver which expresses Gal4 specifically in glial cells at all developmental stages. We crossed UAS-d5-HT_7_R virgins or UAS-h5-HT_7_R virgins with UAS-GFP; *repo-Gal4/TMSbTb* males. UAS-5-HT_7_R /UAS-GFP; *repo-Gal4* were obtained in expected number (50%), indicating that expression of 5-HT_7_R in glial cells was not lethal. However, the F1 *repo>UAS-5-HT_7_R* have a reduced lifespan. (Figure 3). The average life (50% mortality) is smaller when expressing d5-HT_7_R (26 days) or h5-HT_7_R (31 days) in glial cells when compared to the *repo>GFP* control (48 days) or UAS controls (57 days).

These results suggest that 5-HT_7_R expression (i) can lead to reducing cell proliferation (ii) can interfere with EGFR signaling and (iii) does not induce lethality when expressed in glial cells, underlying the interest of evaluating the role of 5-HT_7_R in glioma progression.

### 3.3. Expression of h5-HT_7_R in a Glioma Model Partly Suppress Larval Lethality

**Figure 4:**
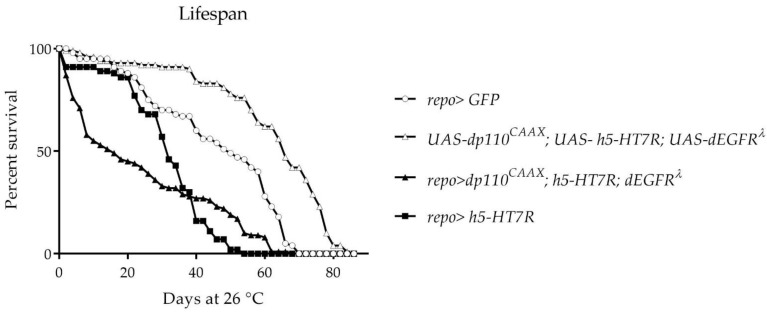
Lifespan of escaper flies. Results correspond to the percentage of living flies *repo>dp110^CAAX^*; h5-HT_7_R; dEGFR^λ^ as a function of time at 26 °C. Lifespan of controls corresponding to the driver *repo>GFP* alone and UAS-h5-HT_7_R or UAS-110^CAAX^, h5-HT_7_R; dEGFR^λ^ alone is also shown.

We then assessed the effect of h5-HT_7_R expression in glioma development. We decided to use the human transgene for two reasons: first, phenotypes induced by expression of human transgene in wings are very similar with those of Drosophila receptor; secondly, this model will offer an opportunity to evaluate the effect of 5-HT_7_R ligands which have been well characterized on the human form of the receptor. To obtain a glioma model in Drosophila, we generated the model developed by Read et al. [57] obtained by co-expression of constitutive forms of EGFR (dEGFR^λ^) and PI3K (dp110^CAAX^) in glial cells using the Gal4-repo driver. This leads to hyperproliferation of glial cells and lethality before the adult stage. We constructed a Drosophila line that contains, in addition to transgenes allowing glioma development (UAS-dp110^CAAX^ and UAS-dEGFR^λ^), a transgene corresponding to h5-HT_7_R. This line was crossed with UAS-GFP; *repo-Gal4/TMSbTB*. While no *repo-Gal4* adults F1 were obtained in the glioma model [57], some escapers arise when expressing h5-HT_7_R since *repo-Gal4* F1 were observed. However, these F1 are in lower number than expected (25% versus 50%), suggesting that some of them died before the adult stage. The lifespan of the survivors was investigated (Figure 4). It appears that survivors (*repo>dp110^CAAX^*, h5-HT_7_R; dEGFR^λ^) can be classified into two groups, those whose lifespan does not exceed 10 days (50% of F1 escapers) which corresponds to an excess of early deaths in the population and those whose lifespan is increased, the survivorship curve declines slowly for a long period from 18 to 60 days.

**Figure 5:**
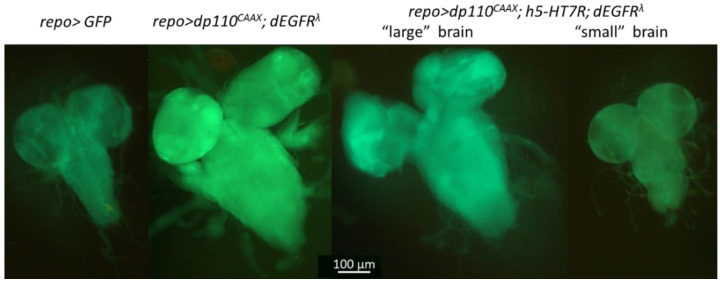
h5-HT_7_R expression reduces proliferation in glioma. Fluorescence microscopy images of third instar larval brains. Larvae expressing h5-HT_7_R in a glioma context (*repo>dp110^CAAX^*; h5-HT_7_R; dEGFR^λ^) exhibit brains of different sizes: “large” brains look like glioma (*repo>dp110^CAAX^*; dEGFR^λ^), “small” brains look like controls (*repo>GFP*). Green fluorescent protein (GFP) is expressed in glial cells.

In order to investigate whether these two groups were the consequence of difference in glioma development, we looked at the morphology of brains at the third instar larvae. Among *repo>dp110^CAAX^*; h5-HT_7_R; dEGFR^λ^ larvae, two classes can be distinguished: those whose brain was similar in size to the glioma model (“large” brains) and those whose brain was more similar to the control (“small” brains) (Figure 5). These results indicate that expression of h5-HT_7_R in Drosophila glioma cells partly rescues flies from lethality. We checked that h5-HT_7_R was indeed expressed in these two categories of brains by Western blots analysis (Figure S2). However, some larvae still have a brain morphology corresponding to excessive cells proliferation as observed in the glioma model (*repo>dp110^CAAX^*; dEGFR^λ^). We then decided to better characterize these two types of brains (“large” and “small”) in order to assess whether the “small” brains had characteristics that were more consistent with controls and the “large” brains with glioma.

### 3.4. Expression of h5-HT_7_R Modifies Glioma Metabolism

**Figure 6:**
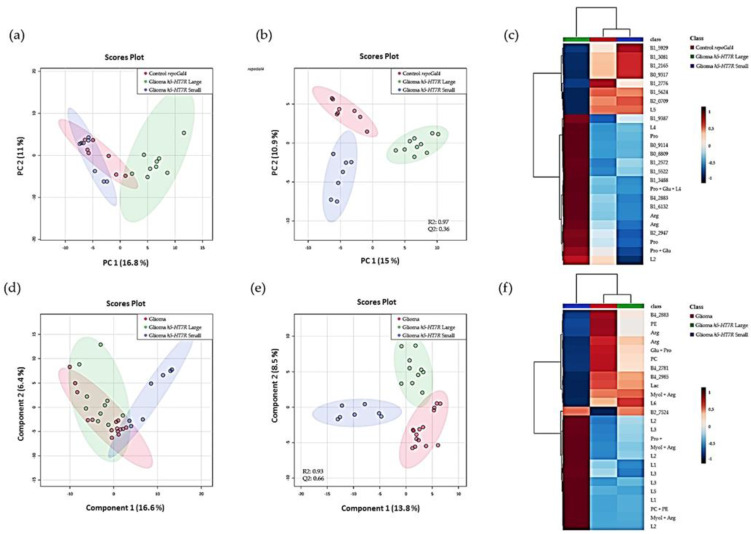
HR-MAS based metabolomics results. (**a**–**c**) Comparison of controls brains (*repo-Gal4*), and brains expressing h5-HT_7_R in a glioma context (“small” brains and “large” brains). (**d**–**f**) Comparison of gliomas and brains expressing h5-HT_7_R in a glioma context (“small” and “large” brains). (**a**,**d**) Unsupervised multivariate analysis PCA, (**b**,**e**) PLS–DA score plot, (**c**,**f**) Heat map of the first 25 most significant buckets with group averaging. R2 is the percentage of the data explained by the supervised model and Q2 is the quality of the prediction. Arg: arginine; Glu: glutamate; Lac: lactate; MyoI: myoinositol; PC: phosphocholine; PE: phosphoethanolamine; Pro: proline; L1, L2, L3, L4, L5 and L6: lipids. L1: CH_3_; L2: (CH_2_)_n_; L3: CH_2_**-**CH_2_-CO; L4: CH_2_**-**C=; L5: CH**_2_-**CO; L6: CH=CH.

Recently, we showed that the Drosophila glioma model used in this study exhibits alteration of metabolism [66]. Such a modification of metabolism is crucial for cancer cells to assure their proliferation. In this study, we used High-Resolution Magic Angle Spinning nuclear magnetic resonance (HRMAS-NMR) techniques to obtain metabolic profiles in the third instar larval brains. We first compared metabolic profile of “small” and “large” brains with controls. Statistical analyses show a clear separation between groups. PCA score plots (Figure 6a) showed a clustering of “small” brains and control brains groups whereas “large” brains group is separated from these two groups. This observation was confirmed by targeted multivariate analysis. First, the partial least squares–discriminant analysis (PLS–DA) was performed on the three groups. The score plot showed a good separation of the groups (Figure 6b); however, the Q2 of 0.36 indicated that the prediction was moderately accurate. This probably means that two out of the three groups were too similar for the model to perform well. To investigate this, PLS–DA were performed on each couple of groups (Figure S3a,b). The model built for “large” brains and control brains was highly predictive (Q2 = 0.78), while the prediction between “small” brains and Gal4 control was only moderately predictive (Q2 = 0.43). The heat map confirmed that controls and “small” brains were close to each other while “large” brains were more distant (Figure 6c). These results clearly demonstrated that the two types of brains expressing h5-HT_7_R in a glioma context had different metabolic fingerprints, the” small” brains being very similar to the Gal4 control brains. It was then interesting to know if the metabolic profile of “large” brains was similar to that of gliomas. For this purpose, we performed the same statistical analyses on glioma expressing h5-HT_7_R and glioma to see if the expression of h5-HT_7_R had an effect on glioma metabolism. PCA score plot showed a clear grouping of “large” brains and glioma and a partial separation of these two groups from the “small” brains along the first Principal Component (PC1 = 15 %) (Figure 6d). A prediction model was successfully built but was only moderately predictive (Q2 = 0.66) (Figure 6e). As before, PLS–DA was performed on each couple of groups to verify which groups are similar (Figure S3c,d). The model built for “large” brains and glioma was moderately predictive (Q2 = 0.50), while the prediction between “small” brains and glioma was highly predictive (Q2 = 0.80). The heat map on the group averages of the top 25 buckets confirmed that the “large” brains and glioma had a close metabolic fingerprint while the “small” brains was different (Figure 6f). On a metabolic level, all these results combined seemed to suggest, that the “small” brains lost characteristics of gliomas and reached a “normal state” during development while the “large” brains are still highly similar to glioma. We then looked at the buckets for which the variable importance in projection (VIP) from the PLS–DA was greater than 1. In total 85 VIPs allowed to identify arginine, valine, glutamine, myoinositol, lactate, phosphocholine (PC) and phophoethanolamine (PE) as metabolites allowing discrimination between the two classes of glioma brains expressing h5-HT_7_R and control brains. All these metabolites have already been described as discriminating glioma brains from control brains [66]. These results suggest that h5-HT_7_R expression may alter glial cell metabolism and thus affect the growth of glioma.

**Figure 7:**
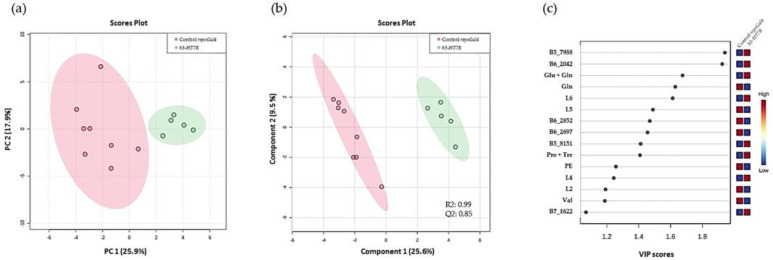
HR-MAS based metabolomics results. Comparison of controls brains (*repo-Gal4*) and brains expressing h5-HT_7_R alone. (**a**) Unsupervised multivariate analysis PCA, (**b**) score plot of the PLS–DA of the *repo-Gal4* control brains and the brains expressing h5-HT_7_R. The Q2 indicates the quality of the prediction, (**c**) VIP scores for the first 15 buckets and their relative concentration in the two groups. Glu: glutamate; Gln: glutamine; PE: phosphoethanolamine; Pro: proline; Tre: trehalose; Val: valine. L2, L4, L5 and L6: fatty acids. L2: (CH_2_)_n_; L4: CH_2_-C=; L5: CH_2_-CO; L6: CH=CH. Red: high level, blue: low level.

Furthermore, the expression of h5-HT_7_R may also have an effect on the metabolism of healthy larval brains. To address this point, we compared brains expressing h5-HT_7_R alone and control brains. The PCA score plot showed a separation of the two groups along the PC1 axis (Figure 7a). The prediction model built with PLS–DA was highly predictive (Q2 = 0.85) which suggest that the expression of h5-HT_7_R alone induced changes in metabolic profile (Figure 7b). In order to identify these alterations, we looked at the variable importance in projection (VIP) which were greater than 1. In total, there were 15 buckets with a VIP > 1, 10 of which had a *p*-value < 0.05 after a t-test with false discovery rate (FDR) correction (Figure 7c). Most of these buckets could not be identified unambiguously, except for those corresponding to glutamine, valine, phosphoethanolamine and lipids. In h5-HT_7_R-expressing brains, we observed a decrease in glutamine, suggesting impaired glutamate/glutamine recycling mediated by glial cells. Usually, glial cells uptake glutamate in synapses via the dEAAT1 receptor and recycle it into glutamine. Interestingly the overlapping glutamate + glutamine signal appears higher in h5-HT_7_R-expressing brains suggesting that glutamate would significantly increase reinforcing the idea that one of the functions of glial cells function is affected. An excess of glutamate could explain the reduced lifespan of adults expressing h5-HT_7_R. Indeed, glutamate is a neurotransmitter which, when accumulated in synapses, is excitotoxic. We also observed an increase in unsaturated fatty acids (5.32 ppm and 2.03 ppm) in brains expressing h5-HT_7_R, suggesting a modification in metabolism of lipids. Phosphoethanolamine, involved in biosynthesis of phosphatidylethanolamine, a major constituent of membrane cells, particularly in brain, appears decreased. As a whole, our results demonstrate that expressing h5-HT_7_R in healthy glial cells affects brain metabolism.

### 3.5. Expression of h5-HT_7_R Modifies Glioma Epigenetic Pattern

**Figure 8:**
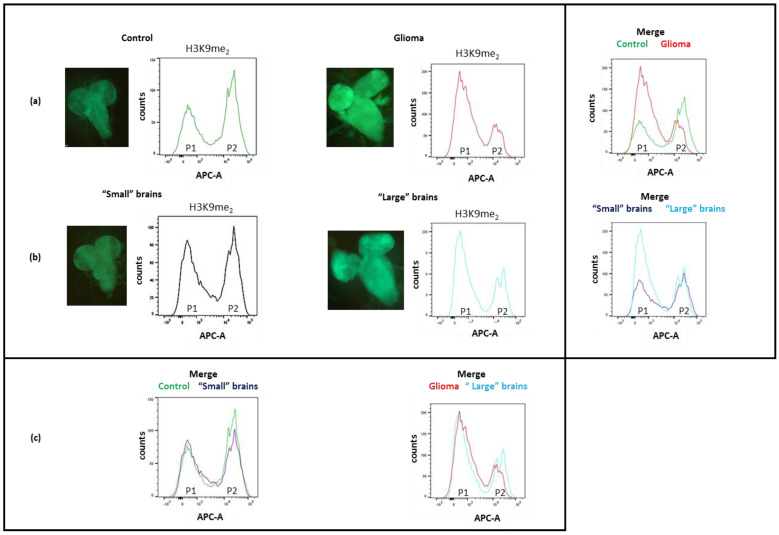
Analysis of H3K9me2 by flow cytometry in glial cells (with GFP expression). Pattern of H3K9me_2_ in glial cells is shown for controls (*repo>GFP*), glioma (*repo>dp110^CAAX^*; dEGFRλ), “small” and “large” brains (*repo>dp110^CAAX^*; h5-HT_7_R; dEGFR^λ^). Two populations of glial cells P1 and P2 differing by their methylation rate have been identified. (**a**) Comparison between controls and gliomas. (**b**) Comparison between “small” and “large” brains. (**c**) Comparison between “small” brains and controls and between “large” brains and gliomas.

Metabolism and epigenetic processes are intimately linked and epigenetic marks are strongly affected in cancer cells [69]. Thus, we have investigated epigenetic marks of glial cells first in the glioma model. We focused on three epigenetic marks: H3K4me2, H3K4me3 and H3K9me2. H3K4me2/me3 are associated with transcriptional activation in opposition with H3K9me_2_/me_3_ that are associated with transcriptional inactivation [70]. We used flow cytometry to measure H3K4 and H3K9 methylation, specifically in glial cells, taking advantage of the fact that, in our model, only glial cells expressed GFP. Each sample was labelled with antibodies directed against H3K9me2, H3K4me2 or H3K4me3 and in parallel with an antibody directed against H3 as a control for H3 content (Figure S4). Results obtained with anti H3K9me2 are presented on Figure 8. Surprisingly, two populations of glial cells, P1 and P2, are observed in controls and in gliomas, while a single population is detected with antibodies against H3 (Figure 8a and Figure S4). This suggests that two populations of glial cells, differing by their H3K9 methylation rate co-exist in larval brains. Similar results are observed for H3K4me2 and H3K4me3 (Figure S4). These marks have been involved in cell lineage commitment and reprogramming of somatic cells in pluripotent cells. Increase in H3K4 or H3K9 methylation is associated with differentiation [71,72,73].

In controls, P2 is the major population. On the contrary, in gliomas P1 is the major one. Whatever the H3 methylation studied (H3K4 or H3K9) the less methylated population increased in gliomas, indicating that not all glial cells proliferate to form the glioma but only one class of glial cells, those with the lowest level of methylation. This class probably corresponds to not fully differentiated cells. This is in agreement with Read et al. [57] who have shown that expression of the constitutive forms of PI3K and EGFR in a subset of differentiated glial cells had no effect on glial cell proliferation leading them to propose that only glial precursors participate in glioma formation as in human.

Then, we focused on the methylation rate of H3K9 in “large” and “small’ brains (*repo>dp110^CAAX^*; h5-HT_7_R; dEGFR^λ^). If “small” brains recovered to a normal state, they should present an identical profile to that of the control. In contrast, “large” brains, if not rescued by h5-HT_7_R expression, should show a H3K9me2 pattern similar to that of gliomas. Comparison of “small” and “large” brains shows that the two spectra differ in a similar way to controls and gliomas (Figure 8b). The superposition of “small” brains and control profiles clearly shows that “small” brains are identical to controls. On the contrary, gliomas and “large” brains profiles do not completely overlap (Figure 8c). The P2 population in “large” brains is increased as compared to gliomas and is more like that of control or “small” brains. Considering H3K9me2, the results suggest that “small” brains have been developed as normal brains, while large brains have been only partly rescued by h5-HT_7_R expression as they present both normal and glioma features

All these results show that expression of 5-HT_7_R partly rescues glioma at the metabolic and epigenetic levels. We then wanted to investigate which signaling pathways may be involved in 5-HT_7_R effects.

### 3.6. cAMP Level Is Decreased in Drosophila Glioma Model

**Figure 9:**
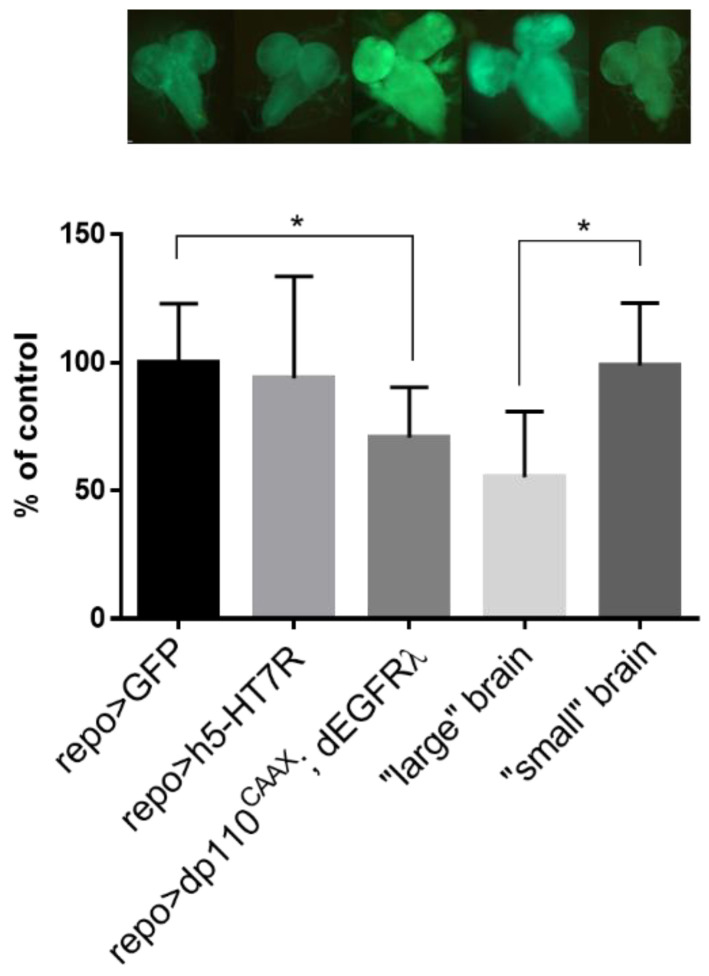
Measurement of cAMP levels has been performed in brains of controls (*repo>GFP*), glioma (*repo>dp110^CAAX^*; dEGFR^λ^) and expressing h5-HT_7_R alone (*repo>h5-HT_7_R*) and in “small” and “large” brains (*repo>dp110^CAAX^*; h5-HT_7_R; dEGFR^λ^). Values have been normalized with the concentration of proteins detected in each sample and expressed as a percentage of control. The upper panel represents images of fluorescent brains for each associated condition and observed by fluorescence microscopy. *, *p*< 0.05. Statistical analysis was done by one-way ANOVA using Tukey’s multiple comparison test, n = 8.

5-HT_7_R is coupled to Gs/cAMP pathway and its activation produce cAMP, a second messenger known to modulate various physiological processes [74]. In particular, cAMP may exhibit opposing effects on cell proliferation depending of the cell type [75]. Several studies have shown that in some cancers, a reduction in cAMP is associated with malignancy, particularly in brain tumors [76]. Gliomagenesis has been related to suppression of cAMP [77,78,79]. Thus, we measured cAMP levels in gliomas and control brains. As expected, glioma exhibited a lower cAMP level than controls (Figure 9). When we compared the cAMP level in “large” and “small” brains, we observed a significant difference between the groups. Indeed, cAMP levels detected in “large” brains and glioma were very similar, whereas levels detected in “small” brains were not significantly different from the controls (Figure 9). In addition, the cAMP level in brains expressing only h5-HT_7_R does not appear different from the controls. Therefore, as previously observed at the level of metabolic and epigenetic profiles, we also detected a difference in cAMP levels between the two types of brains, the “small” and “large” ones expressing h5-HT_7_R in a glioma context.

### 3.7. h5-HT_7_R Expression Does Not Affect Phosphorylation of AMPK, 4eBP1, S6K

**Figure 10:**
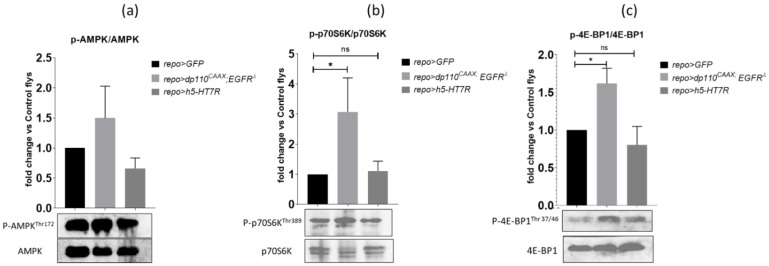
Evaluation of phosphorylation of AMPK, p70S6K and 4E-BP1 in glioma and in brains expressing h5-HT_7_R alone. Western blots of larval brains of controls (*repo>GFP*), glioma (*repo>dp110^CAAX^*; dEGFR^λ^) and expressing h5-HT_7_R alone (*repo>h5-HT_7_R*) probed with (**a**) p-AMPK^Thr172^ and AMPK antibodies, (**b**) p-p70S6K^Thr389^ and p70S6K antibodies and (**c**) p-4E-BP1^Thr37/46^ and 4E-BP1 antibodies. Quantification of mean fold change in phosphorylation was analyzed by one-way ANOVA, Kruskal–Wallis test, Dunn’s multiple comparisons test, n = 6. *, *p* < 0.05. ns, non significant (*p* > 0.05).

We then investigated the downstream cAMP-dependent signaling pathway. cAMP is known to activate the 5′-AMPK-activated kinase (AMPK), a kinase which plays a major role in regulating cellular energy and is involved in brain tumors and gliomas. Indeed, AMPK has been shown to negatively regulate the Warburg effect, thus leading to a decrease in tumor growth [22]. AMPK activation has also been shown to be controlled by cAMP and some GPCRs [80]. Therefore, we sought to determine whether AMPK phosphorylation was affected in glioma and in h5-HT_7_R expressing brains, by immunoblotting using phospho-specific AMPK-Thr172 antibodies. First, we assessed the AMPK phosphorylation status in gliomas (Figure 10a). Results show that there is no significant difference in the level of AMPK threonine phosphorylation between gliomas and controls, although this level tends to be higher in gliomas. Then, we analyzed AMPK phosphorylation at Thr172 in brains expressing h5-HT_7_R alone, but results suggest that h5-HT_7_R expression did not exert any effect on AMPK phosphorylation of AMPK (Thr172).

In parallel, we assessed phosphorylation of the protein kinase p70-S6K and of the eukaryotic initiation factor 4E-binding protein 1 (4E-BP1). These two proteins are effectors of the mTOR signaling pathway which is activated in our Drosophila glioma model. Several studies have shown close relationships between AMPK and mTOR, AMPK suppresses mTORC1, particularly in the brain [10], and 5-HT_7_R is known to activate the mTOR pathway [81]. Thus, phosphorylation of p70S6K and 4E-BP1 was investigated by immunoblotting (Figure 10b,c) in gliomas and brains expressing h5-HT_7_R alone. As expected, phosphorylation of p70S6K and 4E-BP1 is increased in gliomas as compared to controls. In brains expressing h5-HT_7_R alone, no change in the rate of phosphorylation was observed. These results suggest that h5-HT_7_R does not proceed via AMPK or mTOR pathways.

## 4. Discussion

The aim of our work was to assess the role of the serotonin 5-HT_7_ receptor on glioma development by using a Drosophila model.

The 5-HT_7_ receptor is a GPCR positively coupled to adenylate cyclase through activation of Gs, resulting in intracellular increase in cAMP [29,30,31]. Its role in brain tumors has been previously investigated. Mahe et al. showed that in several glioblastoma cell lines, 5-HT_7_R can be detected [40]. Activation of this receptor by serotonin in the U-373 MG astrocytoma cell line induces IL-6 secretion, facilitating tumor progression [41]. Therefore, these results suggest that 5-HT_7_R is associated with tumor progression. In contrast, the present transcriptomic data obtained from human samples show that loss of 5-HT_7_R expression has been observed in several brain tumor types such as astrocytoma, oligodendroglioma and GBM. In addition, the overall survival of patients expressing a low level of the 5-HT_7_R was significantly lower than that of patients with high 5-HT_7_R, suggesting that the 5-HT_7_R expression exerts beneficial effect for tumor regression. In order to explore the role of 5-HT_7_R in the pathogenesis of glioma, we evaluated whether its expression may modify GBM-like phenotypes.

### 4.1. Expression of h5-HT_7_R Partly Reduced Glioma

The Drosophila glioma model used in this study induces lethality; no adults emerge. Interestingly, when h5-HT_7_R is expressed in glioma cells, we obtained adult escapers. Most survivors have a reduced lifespan, and it is rare for them to exhibit a normal lifespan. When observed after dissection, some of the third instar larvae had “large” brains, similar to those of glioma group, and others had “small” brains, a phenotype similar to control group. We hypothesized that the surviving adults come from the larvae with “small” brains. Studies of H3K9me2 epigenetic mark, or cAMP levels in these “small” brains indicate that they rather look like control brains. However, studies of metabolic profiles reveal that “small” brains, although close to controls, are not identical, suggesting that “small” brains have not completely recovered a “normal” state. This may explain why adult escapers have shorter lifespans compared to the controls. The same metabolic biomarkers have been studied in “large” brains and the results showed that their profiles are very close to gliomas, however not completely identical, suggesting that h5-HT_7_R expression can partially reduce glioma cells phenotype.

These results suggest that 5-HT_7_R expression can more or less effectively rescue lethality and glial cell over-proliferation induced by expression of constitutive forms of PI3K and EGFR in larvae. When expressed in glial cells of adult brain the two constitutive forms of PI3K and EGFR also induced hyperproliferation of glial cells as in larvae [82]. It would be particularly relevant to address the question of whether the effect observed here, in a larval glioma model, is transposable to adult glioma model. Drosophila 5-HT_7_R is normally expressed in larval and adult brains and plays roles in learning and behavior [83,84]. However, we cannot exclude that expression of 5-HT_7_R in adult glial cells affects different signaling pathways or different mechanisms, adult glial cells being probably not in the same state of competence as the larval glial cells.

### 4.2. Molecular Mechanisms Involved in the Rescue of Gliomas by h5-HT_7_R

Serotonin is a biogenic monoamine that is expressed in various tissues and has different functions. It is the ligand of numerous serotonin receptors which are related to various signaling pathways. Serotonin has been shown to have a growth stimulatory effect on several types of tumor cells [26]. However, this effect is mainly observed only for high concentrations. At physiological concentrations, serotonin might instead limit tumor growth by acting on vascularization. Here, we have shown that 5-HT_7_ receptor acts as a suppressor of a tumor in a Drosophila glioma model. Similarly, the human 5-HT1B receptor has also been shown to act as a tumor suppressor during lung, renal, oral, osteo carcinogenesis and non-Hodgkin lymphomas. Klempin et al. [85] demonstrated that serotonin receptors may have an opposite effect on hippocampal neurogenesis. The 5-HT1a receptor increases cell proliferation in vivo. On the contrary, the 5-HT2 receptor decreases cell proliferation. This shows that the effect of serotonin and its receptors is complex and depends on different parameters such as the nature of the receptor, the type of cells and the genetic context.

Several studies have revealed that serotonin might also affect cell differentiation. Morita et al. [86] have shown that treatment of rat C6 glioma with serotonin could induce differentiation of the cells. As suggested by our observations and those of Read et al. [62] only glial progenitors would participate to glioma. Expression of h5-HT_7_R in these cells could mimic serotonin activation and induce their differentiation. Consequently, these cells would no longer be competent to hyperproliferate. However, this would not explain why we observe a defect in metabolism of glutamate/glutamine, except if the function of glial cells is simultaneously altered. Müller et al. have already shown that another serotonin receptor (5-HT4R) may modulate astrocyte morphology and function [87].

As 5-HT_7_R is coupled to Gs/cAMP pathway, the tumor suppressor effect of h5-HT_7_R in our glioma model could also be mediated by cAMP. Previous studies have already demonstrated that manipulating the cAMP level disturbs glioma growth and glial cell differentiation. Therefore, cyclic AMP activators have been shown to reduce glioma growth [88,89]. In contrast, a decrease in cAMP levels participates to gliomagenesis in NF-1 mouse model [78]. cAMP is also involved in regulation of AMPK [90]. AMPK is a crucial protein which regulates multiple pathways and is particularly important in regulating metabolism. It acts as a sensor of cellular energy. AMPK activation has been implicated in tumorigenesis, but it can also promote anti-tumor responses [91]. Faubert et al. [22] have shown that AMPK suppresses tumor growth by negatively regulating the Warburg effect. In the case of glioblastoma it seems to act as pro-tumorigenic [92]. Here, as already observed in some cancers [78], we found that cAMP level was decreased in glioma. Although 5-HT_7_R is coupled to Gs/cAMP pathway, we did not detect any significant cAMP increase in brains expressing h5-HT_7_R alone, as one would expect. We speculated that this lack of increase might be related to the samples we used for the cAMP assays. Indeed, we used samples from whole brains, in which glial cells represent only 10% of total cells. Therefore, a small change in these subtypes of cells would not be enough to be detected in our assays. Alternatively, the lack of 5-HT_7_R effect on cAMP may be explained by a low level of 5-HT_7_R activation-induced by endogenous serotonin and/or a low level of constitutive activity. It is well known that 5-HT_7_R, like other serotoninergic receptors when expressed in a heterologous system, can display a high constitutive activity, thereby enhancing its functional coupling to G_S_ signaling and cAMP synthesis [93]. This property corresponds to the ability of a given receptor to be spontaneously active without agonist stimulation. Such a constitutive activity has been demonstrated by using 5-HT_7_R ligands acting as inverse agonist on adenylate cyclase activity and able to block this basal activation [94,95,96]. However, the existence of 5-HT_7_R constitutive activity in native tissue remain an open question. In Drosophila, even if 5-HT_7_R exhibit a constitutive activity in glial cells, it is probably too small to be detected in our experimental conditions. Similarly, we did not observe any effect on AMPK phosphorylation at Thr171. Phosphorylation at this site allows AMPK activation which is dependent of the kinase LKB1. Interestingly, Read et al. [97] used a RNAi screen in Drosophila glial cells and have shown that expressing RNAi targeting LKB1 has no effect on glial neoplasia. This could indicate that AMPK is not essential to glial cell proliferation induced by expression of constitutive EGFR and PI3K proteins. Other phosphorylation sites have been identified, in particular serine 491, which, when phosphorylated, inhibits AMPK activity. Several protein kinases have been identified that phosphorylate Ser491, in particular p70S6K which is activated in our model.

### 4.3. Impact of h5-HT_7_R on Metabolism

Cancer cells exhibit reprograming of their metabolism. It is essential as they must be able to capture nutrients in the external environment in a significant way to produce ATP, to synthesize macromolecules (proteins, lipids, nucleic acids) and their precursors to ensure their proliferation and to tolerate oxidative stress or hypoxia, for example. Metabolic changes in cancer cells, therefore, have a very significant impact not only in ensuring the biomass necessary for their own proliferation, but also in regulating gene expression via epigenetic processes [98]. Unravelling this altered metabolism may be disadvantageous for proliferation of cancer cells. Metabolomic analysis of larval brains has clearly shown that metabolism was affected by expression of h5-HT_7_R alone. In particular, metabolism of the two amino acids glutamate and glutamine, but also metabolism of lipids and phosphoethanolamine. It is now established that fatty acids are critical bio-energetic substrates within the glioma cells [99]. They use fatty acids as a substrate for energy production and inhibition of fatty acid synthesis reduces proliferation of glioma cells [100,101]. Fatty acids also play important roles in forming phospholipids involved in plasma membranes and glycerophospholipids. Phosphoethanolamine also plays an important role in cancer cells. Osawa et al. (2019) found that accumulation of phosphoethanolamine (PEtn) protects cancer cells [102]. In addition, they show that glutamine regulates PE biosynthesis. Interestingly, when expressing h-5HT7R alone in glial cells, we observed a decrease in phosphoethanolamine and glutamine. In a glioma context, these effects may adversely impact tumor growth.

Some of our results also suggest that expression of 5-HT_7_R could interfere with EGFR signaling. Indeed, when expressing 5-HT_7_R in wings, induced phenotypes were reminiscent of a reduction in cellular proliferation and of EGFR signaling. When 5-HT_7_R was expressed throughout development in glial cells, adults exhibited a shorter lifespan which is one of the characteristics of neurodegeneration. It has been shown that EGFR plays a critical role in glial cell survival during embryonic development [103]. In addition, EGFR knock-out mice contain lower numbers of GFAP positive astrocytes in cortex and develop progressive neurodegeneration in the frontal cortex, olfactory bulb and thalamus [104]. We may expect that if 5-HT_7_R is a negative regulator of EGFR signaling, then there is a lower number of glial cells in brains expressing the receptor and consequently neurodegeneration. In a recent study, we investigated whether 5-HT_7_R activation can induce transactivation of EGFR. However, when using an EGFR inhibitor, we did not observe any change in 5-HT_7_R signaling (paper in revision). Further investigations are needed to evaluate the reciprocal effect, whether the modulation of 5-HT_7_R activity can affect EGFR signaling. Functional crosstalk between 5-HT_7_R and EGFR may also regulate EGFR internalization, and therefore, downstream signaling including MAPK cascade and known to be involved in cancer cell proliferation [105]. Alternatively, 5-HT_7_R can crosstalk with EGFR by directly interacting with EGFR, as proposed for other GPCR, underlying the importance of protein–protein interaction to modulate cancer cell signaling [106].

By negatively regulating EGFR signaling, h5-HT_7_R may also modify the metabolism of glial cells. EGFR pathway has been involved in several critical metabolic processes in cancer cells, such as biosynthesis of fatty acids and glucose catabolism [107,108,109,110]. Thus, decreasing EGFR signaling should allow to recover a “normal” metabolism, and therefore, imped cellular proliferation.

## 5. Conclusions

In conclusion, we have confirmed a functional role of 5-HT_7_ receptor as a tumor suppressor that controls glioma development. Its expression in a Drosophila glioma model results in a decrease in larval lethality associated with the presence of surviving adults and a return to normal brain morphology. Furthermore, we demonstrated that 5-HT_7_R can restore molecular markers affected in gliomas. Several hypotheses can be proposed to explain the role of 5-HT_7_R on the development of gliomas: (i) 5-HT_7_R is known to be coupled to Gs/cAMP pathways, we suggest that changes in cAMP levels may participate in the switch of metabolic state and cell differentiation; (ii) 5-HT_7_R may also acts by modifying metabolism, as we observed metabolic alterations induced by 5-HT_7_R; and (iii) 5-HT_7_R can interfere with EGFR signalling. It is likely that multiple signaling pathways are involved in 5-HT_7_R beneficial effect on tumor growth. Altogether, our results suggest that 5-HT_7_R may be considered as an interesting target for the treatment of glioma. The glioma model in Drosophila may be useful for drug screening and for the characterization of new 5-HT_7_R ligands for brain tumor therapy.

## Data Availability

Not applicable.

## Supplementary Materials

The following supporting information can be downloaded at: https://www.mdpi.com/article/10.3390/cells11081281/s1. Table S1: List of antibodies used in flow cytometry experiments; Figure S1: Flow cytometry controls. White cells and isotypic controls in gliomas and control brains; Figure S2: Expression of h5-HT_7_R in “small” and “large” brains expressing h5-HT_7_R (*repo>dp110^CAAX^; h5-HT_7_R; dEGFR^λ^*). Controls are *w^1118^* brains. Antibodies directed against the FLAG tag were used; Figure S3: PLS–DA score plots. (a) Comparison of controls brains (*repo-Gal4*), and “small” brains expressing *h5-HT_7_R* in a glioma context, (b) Comparison of controls brains (*repo-Gal4*), and “large” brains expressing *h5-HT_7_R* in a glioma context. (c) Comparison of gliomas and “small” brains expressing *h5-HT_7_R* in a glioma context, (d) Comparison of gliomas and “large” brains expressing *h5-HT_7_R* in a glioma context; Figure S4: Analysis of H3 content and H3K4me2/3 by flow cytometry in glial cells (GFP labelled cells). Pattern of H3 labelling and H3K4me2/3 labelling in glial cells are shown for controls (*repo>GFP*) and glioma (*repo>**dp110^CAAX^; dEGFR^λ^*). Two populations of glial cells P1 and P2 differing by their methylation rate have been identified

## References

[1] Louis D.N., Perry A., Reifenberger G., Von Deimling A., Figarella-Branger D., Cavenee W.K., Ohgaki H., Wiestler O.D., Kleihues P., Ellison D.W. (2016). The 2016 World Health Organization Classification of Tumors of the Central Nervous System: A summary. Acta Neuropathol..

[2] Giaccone G. (1995). New drugs in non-small cell lung cancer. An overview. Lung Cancer.

[3] Ren H., Yang B.F., Rainov N.G. (2007). Receptor tyrosine kinases as therapeutic targets in malignant glioma. Rev. Recent Clin. Trials.

[4] Zahonero C., Sánchez-Gómez P. (2014). EGFR-dependent mechanisms in glioblastoma: Towards a better therapeutic strategy. Cell Mol. Life Sci..

[5] Kondo Y., Katsushima K., Ohka F., Natsume A., Shinjo K. (2014). Epigenetic dysregulation in glioma. Cancer Sci..

[6] Hashizume R. (2017). Epigenetic Targeted Therapy for Diffuse Intrinsic Pontine Glioma. Neurol. Med. Chir..

[7] Zang L., Kondengaden S.M., Che F., Wang L., Heng X. (2018). Potential Epigenetic-Based Therapeutic Targets for Glioma. Front. Mol. Neurosci..

[8] de Souza C.F., Sabedot T.S., Malta T.M., Stetson L., Morozova O., Sokolov A., Laird P.W., Wiznerowicz M., Iavarone A., Snyder J. (2018). A Distinct DNA Methylation Shift in a Subset of Glioma CpG Island Methylator Phenotypes during Tumor Recurrence. Cell Rep..

[9] Liao P., Ostrom Q.T., Stetson L., Barnholtz-Sloan J.S. (2018). Models of epigenetic age capture patterns of DNA methylation in glioma associated with molecular subtype, survival, and recurrence. Neuro-Oncology.

[10] Zhang C., Zhang J., Hao J., Shi Z., Wang Y., Han L., Yu S., You Y., Jiang T., Wang J. (2012). High level of mir-221/222 confers increased cell invasion and poor prognosis in glioma. J. Transl. Med..

[11] Liau B.B., Sievers C., Donohue L.K., Gillespie S.M., Flavahan W.A., Miller T.E., Venteicher A.S., Hebert C.H., Carey C.D., Rodig S.J. (2017). Adaptive Chromatin Remodeling Drives Glioblastoma Stem Cell Plasticity and Drug Tolerance. Cell Stem Cell.

[12] Wang X.-Q., Bai H.-M., Li S.-T., Sun H., Min L.-Z., Tao B.-B., Zhong J., Li B. (2017). Knockdown of HDAC1 expression suppresses invasion and induces apoptosis in glioma cells. Oncotarget.

[13] Ghildiyal R., Sen E. (2017). Concerted action of histone methyltransferases g9a and prmt-1 regulates pgc-1alpha-rig-i axis in ifngamma treated glioma cells. Cytokine.

[14] Sharma S., Kelly T.K., Jones P.A. (2010). Epigenetics in cancer. Carcinogenesis.

[15] Shi W., Palmer J.D., Werner-Wasik M., Andrews D.W., Evans J.J., Glass J., Kim L., Bar-Ad V., Judy K., Farrell C. (2016). Phase I trial of panobinostat and fractionated stereotactic re-irradiation therapy for recurrent high grade gliomas. J. Neuro Oncol..

[16] Ghiaseddin A., Reardon D., Massey W., Mannerino A., Lipp E.S., Herndon J.E., McSherry F., Desjardins A., Randazzo D., Friedman H.S. (2018). Phase II study of bevacizumab and vorinostat for patients with recurrent world health organization grade 4 malignant glioma. Oncologist.

[17] Guo A.-S., Huang Y.-Q., Ma X.-D., Lin R.-S. (2016). Mechanism of G9a inhibitor BIX-01294 acting on U251 glioma cells. Mol. Med. Rep..

[18] Bi J., Chowdhry S., Wu S., Zhang W., Masui K., Mischel P.S. (2020). Altered cellular metabolism in gliomas—An emerging landscape of actionable co-dependency targets. Nat. Rev. Cancer.

[19] Lien E.C., Lyssiotis C.A., Cantley L.C. (2016). Metabolic reprogramming by the pi3k-akt-mtor pathway in cancer. Recent Results Cancer Res..

[20] Warburg O. (1956). On the Origin of Cancer Cells. Science.

[21] Mashimo T., Pichumani K., Vemireddy V., Hatanpaa K.J., Singh D.K., Sirasanagandla S., Nannepaga S., Piccirillo S.G., Kovacs Z., Foong C. (2014). Acetate Is a Bioenergetic Substrate for Human Glioblastoma and Brain Metastases. Cell.

[22] Faubert B., Boily G., Izreig S., Griss T., Samborska B., Dong Z., Dupuy F., Chambers C., Fuerth B.J., Viollet B. (2013). AMPK Is a Negative Regulator of the Warburg Effect and Suppresses Tumor Growth In Vivo. Cell Metab..

[23] Xing F., Luan Y., Cai J., Wu S., Mai J., Gu J., Zhang H., Li K., Lin Y., Xiao X. (2017). The anti-warburg effect elicited by the camp-pgc1alpha pathway drives differentiation of glioblastoma cells into astrocytes. Cell Rep..

[24] Merzak A., Koochekpour S., Fillion M.-P., Fillion G., Pilkington G.J. (1996). Expression of serotonin receptors in human fetal astrocytes and glioma cell lines: A possible role in glioma cell proliferation and migration. Brain Res. Mol. Brain Res..

[25] Balakrishna P., George S., Hatoum H., Mukherjee S. (2021). Serotonin Pathway in Cancer. Int. J. Mol. Sci..

[26] Sarrouilhe D., Clarhaut J., Defamie N., Mesnil M. (2015). Serotonin and cancer: What is the link?. Curr. Mol. Med..

[27] Vicaut E., Laemmel E., Stücker O. (2000). Impact of serotonin on tumour growth. Ann. Med..

[28] Barnes N.M., Sharp T. (1999). A review of central 5-HT receptors and their function. Neuropharmacology.

[29] Bard J., Zgombick J., Adham N., Vaysse P., Branchek T., Weinshank R. (1993). Cloning of a novel human serotonin receptor (5-HT_7_) positively linked to adenylate cyclase. J. Biol. Chem..

[30] Lovenberg T.W., Baron B.M., de Lecea L., Miller J.D., Prosser R., Rea M.A., Foye P.E., Racke M., Slone A.L., Siegel B.W. (1993). A novel adenylyl cyclase-activating serotonin receptor (5-HT_7_) implicated in the regulation of mammalian circadian rhythms. Neuron.

[31] Ruat M., Traiffort E., Leurs R., Tardivel-Lacombe J., Diaz J., Arrang J.M., Schwartz J.C. (1993). Molecular cloning, characterization, and localization of a high-affinity serotonin receptor (5-HT_7_) activating cAMP formation. Proc. Natl. Acad. Sci. USA.

[32] Hedlund P.B., Sutcliffe J.G. (2004). Functional, molecular and pharmacological advances in 5-HT_7_ receptor research. Trends Pharmacol. Sci..

[33] Yaakob N.S., Chinkwo K.A., Chetty N., Coupar I.M., Irving H.R. (2015). Distribution of 5-ht3, 5-ht4, and 5-ht7 receptors along the human colon. J. Neurogastroenterol. Motil..

[34] Quintero-Villegas A., Valdés-Ferrer S.I. (2019). Role of 5-HT_7_ receptors in the immune system in health and disease. Mol. Med..

[35] Brenchat A., Nadal X., Romero L., Ovalle S., Muro A., Sánchez-Arroyos R., Portillo-Salido E., Pujol M., Montero A., Codony X. (2010). Pharmacological activation of 5-HT_7_ receptors reduces nerve injury-induced mechanical and thermal hypersensitivity. Pain.

[36] Nikiforuk A. (2015). Targeting the Serotonin 5-HT_7_ Receptor in the Search for Treatments for CNS Disorders: Rationale and Progress to Date. CNS Drugs.

[37] Zareifopoulos N., Papatheodoropoulos C. (2016). Effects of 5-HT-7 receptor ligands on memory and cognition. Neurobiol. Learn. Mem..

[38] Norum J.H., Hart K., Levy F.O. (2003). Ras-dependent erk activation by the human g(s)-coupled serotonin receptors 5-ht4(b) and 5-ht7(a). J. Biol. Chem..

[39] Kvachnina E., Liu G., Dityatev A., Renner U., Dumuis A., Richter D.W., Dityateva G., Schachner M., Voyno-Yasenetskaya T.A., Ponimaskin E. (2005). 5-HT_7_ Receptor Is Coupled to G alpha Subunits of Heterotrimeric G12-Protein to Regulate Gene Transcription and Neuronal Morphology. J. Neurosci..

[40] Mahé C., Bernhard M., Bobirnac I., Keser C., Loetscher E., Feuerbach D., Dev K.K., Schoeffter P. (2004). Functional expression of the serotonin 5-HT_7_receptor in human glioblastoma cell lines. Br. J. Pharmacol..

[41] Lieb K., Biersack L., Waschbisch A., Orlikowski S., Akundi R.S., Candelario-Jalil E., Hull M., Fiebich B.L. (2005). Serotonin via 5-ht7 receptors activates p38 mitogen-activated protein kinase and protein kinase c epsilon resulting in interleukin-6 synthesis in human u373 mg astrocytoma cells. J. Neurochem..

[42] Zohrabian V.M., Forzani B., Chau Z., Murali R., Jhanwar-Uniyal M. (2009). Rho/ROCK and MAPK signaling pathways are involved in glioblastoma cell migration and proliferation. Anticancer Res..

[43] Lopez-Gines C., Gil-Benso R., Benito R., Mata M., Pereda J., Sastre J., Roldan P., Gonzalez-Darder J., Cerda-Nicolas M. (2008). The activation of erk1/2 map kinases in glioblastoma pathobiology and its relationship with egfr amplification. Neuropathol. Off. J. Jpn. Soc. Neuropathol..

[44] Gautam J., Banskota S., Regmi S.C., Ahn S., Jeon Y.H., Jeong H., Kim S.J., Nam T.-G., Jeong B.-S., Kim J.-A. (2016). Tryptophan hydroxylase 1 and 5-HT_7_ receptor preferentially expressed in triple-negative breast cancer promote cancer progression through autocrine serotonin signaling. Mol. Cancer.

[45] Fatima S., Shi X., Lin Z., Chen G.Q., Pan X.H., Wu J.C., Ho J.W., Lee N.P., Gao H., Zhang G. (2016). 5-hydroxytryptamine promotes hepatocellular carcinoma proliferation by influencing beta-catenin. Mol. Oncol..

[46] Adams M.D., Celniker S.E., Holt R.A., Evans C.A., Gocayne J.D., Amanatides P.G., Scherer S.E., Li P.W., Hoskins R.A., Galle R.F. (2000). The Genome Sequence of Drosophila melanogaster. Science.

[47] Rubin G.M., Spradling A.C. (1982). Genetic Transformation of Drosophila with Transposable Element Vectors. Science.

[48] Potter C.J., Turenchalk G.S., Xu T. (2000). Drosophila in cancer research: An expanding role. Trends Genet..

[49] Dzitoyeva S., Dimitrijevic N., Manev H. (2001). Intra-abdominal injection of double-stranded RNA into anesthetized adult Drosophila triggers RNA interference in the central nervous system. Mol. Psychiatry.

[50] Kang H.-L., Benzer S., Min K.-T. (2002). Life extension in Drosophila by feeding a drug. Proc. Natl. Acad. Sci. USA.

[51] Manev H., Dimitrijevic N., Dzitoyeva S. (2003). Techniques: Fruit flies as models for neuropharmacological research. Trends Pharmacol. Sci..

[52] Nichols C.D. (2006). *Drosophila melanogaster* neurobiology, neuropharmacology, and how the fly can inform central nervous system drug discovery. Pharmacol. Ther..

[53] Bilen J., Bonini N.M. (2005). Drosophila as a Model for Human Neurodegenerative Disease. Annu. Rev. Genet..

[54] Freeman M.R., Doherty J. (2006). Glial cell biology in Drosophila and vertebrates. Trends Neurosci..

[55] Read R.D. (2011). *Drosophila melanogaster* as a model system for human brain cancers. Glia.

[56] Witte H.T., Jeibmann A., Klämbt C., Paulus W. (2009). Modeling Glioma Growth and Invasion in Drosophila melanogaster. Neoplasia.

[57] Read R.D., Cavenee W.K., Furnari F.B., Thomas J.B. (2009). A Drosophila Model for EGFR-Ras and PI3K-Dependent Human Glioma. PLoS Genet..

[58] Brumby A.M., Richardson H. (2005). Using *Drosophila melanogaster* to map human cancer pathways. Nat. Rev. Cancer.

[59] Witz P., Amlaiky N., Plassat J.L., Maroteaux L., Borrelli E., Hen R. (1990). Cloning and characterization of a Drosophila serotonin receptor that activates adenylate cyclase. Proc. Natl. Acad. Sci. USA.

[60] Colas J.F., Launay J.M., Kellermann O., Rosay P., Maroteaux L. (1995). Drosophila 5-HT2 serotonin receptor: Coexpression with fushi-tarazu during segmentation. Proc. Natl. Acad. Sci. USA.

[61] Saudou F., Boschert U., Amlaiky N., Plassat J., Hen R. (1992). A family of Drosophila serotonin receptors with distinct intracellular signalling properties and expression patterns. EMBO J..

[62] Johnson O., Becnel J., Nichols C. (2009). Serotonin 5-HT(2) and 5-HT(1A)-like receptors differentially modulate aggressive behaviors in Drosophila melanogaster. Neuroscience.

[63] Yuan Q., Lin F., Zheng X., Sehgal A. (2005). Serotonin Modulates Circadian Entrainment in Drosophila. Neuron.

[64] Bowman R.L., Wang Q., Carro A., Verhaak R.G.W., Squatrito M. (2017). GlioVis data portal for visualization and analysis of brain tumor expression datasets. Neuro Oncol..

[65] Harzer H., Berger C., Conder R., Schmauss G., Knoblich J.A. (2013). FACS purification of Drosophila larval neuroblasts for next-generation sequencing. Nat. Protoc..

[66] Maravat M., Bertrand M., Landon C., Fayon F., Morisset-Lopez S., Sarou-Kanian V., Decoville M. (2021). Complementary Nuclear Magnetic Resonance-Based Metabolomics Approaches for Glioma Biomarker Identification in a *Drosophila melanogaster* Model. J. Proteome Res..

[67] Jacob D., Deborde C., Lefebvre M., Maucourt M., Moing A. (2017). NMRProcFlow: A graphical and interactive tool dedicated to 1D spectra processing for NMR-based metabolomics. Metabolomics.

[68] Pang Z., Chong J., Zhou G., de Lima Morais D.A., Chang L., Barrette M., Gauthier C., Jacques P.-É., Li S., Xia J. (2021). MetaboAnalyst 5.0: Narrowing the gap between raw spectra and functional insights. Nucleic Acids Res..

[69] Huo M., Zhang J., Huang W., Wang Y. (2021). Interplay among metabolism, epigenetic modifications, and gene expression in cancer. Front. Cell Dev. Biol..

[70] Hyun K., Jeon J., Park K., Kim J. (2017). Writing, erasing and reading histone lysine methylations. Exp. Mol. Med..

[71] Wen B., Wu H., Shinkai Y., Irizarry R.A., Feinberg A.P. (2009). Large histone H3 lysine 9 dimethylated chromatin blocks distinguish differentiated from embryonic stem cells. Nat. Genet..

[72] Ugarte F., Sousae R., Cinquin B., Martin E., Krietsch J., Sanchez G., Inman M., Tsang H., Warr M., Passegue E. (2015). Progressive Chromatin Condensation and H3K9 Methylation Regulate the Differentiation of Embryonic and Hematopoietic Stem Cells. Stem Cell Rep..

[73] Bernstein B.E., Mikkelsen T.S., Xie X., Kamal M., Huebert D.J., Cuff J., Fry B., Meissner A., Wernig M., Plath K. (2006). A Bivalent Chromatin Structure Marks Key Developmental Genes in Embryonic Stem Cells. Cell.

[74] Barnes N.M., Ahern G.P., Becamel C., Bockaert J., Camilleri M., Chaumont-Dubel S., Claeysen S., Cunningham K.A., Fone K.C., Gershon M. (2021). International Union of Basic and Clinical Pharmacology. CX. Classification of Receptors for 5-hydroxytryptamine; Pharmacology and Function. Pharmacol. Rev..

[75] Insel P.A., Zhang L., Murray F., Yokouchi H., Zambon A.C. (2012). Cyclic AMP is both a pro-apoptotic and anti-apoptotic second messenger. Acta Physiol..

[76] Furman M.A., Shulman K. (1977). Cyclic AMP and adenyl cyclase in brain tumors. J. Neurosurg..

[77] Warrington N.M., Woerner B.M., Daginakatte G.C., Dasgupta B., Perry A., Gutmann D., Rubin J.B. (2007). Spatiotemporal Differences in CXCL12 Expression and Cyclic AMP Underlie the Unique Pattern of Optic Glioma Growth in Neurofibromatosis Type 1. Cancer Res..

[78] Warrington N.M., Gianino S.M., Jackson E., Goldhoff P., Garbow J.R., Piwnica-Worms D., Gutmann D., Rubin J.B. (2010). Cyclic AMP Suppression Is Sufficient to Induce Gliomagenesis in a Mouse Model of Neurofibromatosis-1. Cancer Res..

[79] Daniel P.M., Filiz G., Mantamadiotis T. (2016). Sensitivity of GBM cells to cAMP agonist-mediated apoptosis correlates with CD44 expression and agonist resistance with MAPK signaling. Cell Death Dis..

[80] Hutchinson D.S., Summers R.J., Bengtsson T. (2008). Regulation of AMP-activated protein kinase activity by G-protein coupled receptors: Potential utility in treatment of diabetes and heart disease. Pharmacol. Ther..

[81] Speranza L., Giuliano T., Volpicelli F., De Stefano M.E., Lombardi L., Chambery A., Lacivita E., Leopoldo M., Bellenchi G.C., di Porzio U. (2015). Activation of 5-HT_7_ receptor stimulates neurite elongation through mTOR, Cdc42 and actin filaments dynamics. Front. Behav. Neurosci..

[82] Chi K.-C., Tsai W.-C., Wu C.-L., Lin T.-Y., Hueng D.-Y. (2019). An Adult Drosophila Glioma Model for Studying Pathometabolic Pathways of Gliomagenesis. Mol. Neurobiol..

[83] Becnel J., Johnson O., Luo J., Nässel D.R., Nichols C.D. (2011). The Serotonin 5-HT_7_Dro Receptor Is Expressed in the Brain of Drosophila, and Is Essential for Normal Courtship and Mating. PLoS ONE.

[84] Ganguly A., Qi C., Bajaj J., Lee D. (2020). Serotonin receptor 5-HT_7_ in Drosophila mushroom body neurons mediates larval appetitive olfactory learning. Sci. Rep..

[85] Klempin F., Babu H., De Pietri Tonelli D., Alarcon E., Fabel K., Kempermann G. (2010). Oppositional effects of serotonin receptors 5-HT1a, 2, and 2c in the regulation of adult hippocampal neurogenesis. Front. Mol. Neurosci..

[86] Morita K., Arimochi H., Itoh H., Her S. (2006). Possible involvement of 5α-reduced neurosteroids in adrenergic and serotonergic stimulation of GFAP gene expression in rat C6 glioma cells. Brain Res..

[87] Müller F.E., Schade S.K., Cherkas V., Stopper L., Breithausen B., Minge D., Varbanov H., Wahl-Schott C., Antoniuk S., Domingos C. (2021). Serotonin receptor 4 regulates hippocampal astrocyte morphology and function. Glia.

[88] Safitri D., Harris M., Potter H., Yan Yeung H., Winfield I., Kopanitsa L., Svensson F., Rahman T., Harper M.T., Bailey D. (2020). Elevated intracellular camp concentration mediates growth suppression in glioma cells. Biochem. Pharmacol..

[89] Wartchow K., Schmid B., Tripal P., Stadlbauer A., Buchfelder M., Gonçalves C.-A., Kleindienst A. (2021). Treatment with Cyclic AMP Activators Reduces Glioblastoma Growth and Invasion as Assessed by Two-Photon Microscopy. Cells.

[90] Aslam M., Ladilov Y. (2022). Emerging Role of cAMP/AMPK Signaling. Cells.

[91] Vara-Ciruelos D., Russell F.M., Hardie D.G. (2019). The strange case of ampk and cancer: Dr jekyll or Mr hyde?. Open Biol..

[92] Leli N.M., Koumenis C. (2018). Pro-tumorigenic AMPK in glioblastoma. Nat. Cell Biol..

[93] De Deurwaerdère P., Bharatiya R., Chagraoui A., Di Giovanni G. (2020). Constitutive activity of 5-HT receptors: Factual analysis. Neuropharmacology.

[94] Ulsund A.H., Dahl M., Frimurer T.M., Manfra O., Schwartz T.W., Levy F.O., Andressen K.W. (2019). Preassociation between the 5-ht7 serotonin receptor and g protein gs: Molecular determinants and association with low potency activation of adenylyl cyclase. FASEB J..

[95] Andressen K.W., Manfra O., Brevik C.H., Ulsund A.H., Vanhoenacker P., Levy F.O., Krobert K.A. (2015). The atypical antipsychotics clozapine and olanzapine promote down-regulation and display functional selectivity at human 5- HT 7 receptors. Br. J. Pharmacol..

[96] Andressen K.W., Ulsund A.H., Krobert K.A., Lohse M.J., Bunemann M., Levy F.O. (2018). Related gpcrs couple differently to gs: Preassociation between g protein and 5-ht7 serotonin receptor reveals movement of galphas upon receptor activation. FASEB J..

[97] Read R.D., Fenton T.R., Gomez G.G., Wykosky J., Vandenberg S.R., Babic I., Iwanami A., Yang H., Cavenee W.K., Mischel P.S. (2013). A Kinome-Wide RNAi Screen in Drosophila Glia Reveals That the RIO Kinases Mediate Cell Proliferation and Survival through TORC2-Akt Signaling in Glioblastoma. PLoS Genet..

[98] Wong C.C., Qian Y., Yu J. (2017). Interplay between epigenetics and metabolism in oncogenesis: Mechanisms and therapeutic approaches. Oncogene.

[99] Strickland M., Stoll E.A. (2017). Metabolic Reprogramming in Glioma. Front. Cell Dev. Biol..

[100] Grube S., Dünisch P., Freitag D., Klausnitzer M., Sakr Y., Walter J., Kalff R., Ewald C. (2014). Overexpression of fatty acid synthase in human gliomas correlates with the WHO tumor grade and inhibition with Orlistat reduces cell viability and triggers apoptosis. J. Neurooncol..

[101] Lin H., Patel S., Affleck V.S., Wilson I., Turnbull D., Joshi A.R., Maxwell R., Stoll E.A. (2017). Fatty acid oxidation is required for the respiration and proliferation of malignant glioma cells. Neuro-Oncology.

[102] Osawa T., Shimamura T., Saito K., Hasegawa Y., Ishii N., Nishida M., Ando R., Kondo A., Anwar M., Tsuchida R. (2019). Phosphoethanolamine Accumulation Protects Cancer Cells under Glutamine Starvation through Downregulation of PCYT2. Cell Rep..

[103] Hidalgo A. (2002). Interactive nervous system development: Control of cell survival in Drosophila. Trends Neurosci..

[104] Sibilia M., Steinbach J.P., Stingl L., Aguzzi A., Wagner E.F. (1998). A strain-independent postnatal neurodegeneration in mice lacking the EGF receptor. EMBO J..

[105] Tomas A., Futter C.E., Eden E.R. (2014). EGF receptor trafficking: Consequences for signaling and cancer. Trends Cell Biol..

[106] Kilpatrick L.E., Hill S.J. (2021). Transactivation of G protein-coupled receptors (GPCRs) and receptor tyrosine kinases (RTKs): Recent insights using luminescence and fluorescence technologies. Curr. Opin. Endocr. Metab. Res..

[107] Guo D., Prins R.M., Dang J., Kuga D., Iwanami A., Soto H., Lin K.Y., Huang T.T., Akhavan D., Hock M.B. (2009). EGFR Signaling Through an Akt-SREBP-1–Dependent, Rapamycin-Resistant Pathway Sensitizes Glioblastomas to Antilipogenic Therapy. Sci. Signal.

[108] Makinoshima H., Takita M., Saruwatari K., Umemura S., Obata Y., Ishii G., Matsumoto S., Sugiyama E., Ochiai A., Abe R. (2015). Signaling through the phosphatidylinositol 3-kinase (pi3k)/mammalian target of rapamycin (mtor) axis is responsible for aerobic glycolysis mediated by glucose transporter in epidermal growth factor receptor (egfr)-mutated lung adenocarcinoma. J. Biol. Chem..

[109] Lim S.-O., Li C.-W., Xia W., Lee H.-H., Chang S.-S., Shen J., Hsu J.L., Raftery D., Djukovic D., Gu H. (2016). EGFR Signaling Enhances Aerobic Glycolysis in Triple-Negative Breast Cancer Cells to Promote Tumor Growth and Immune Escape. Cancer Res..

[110] Sigismund S., Avanzato D., Lanzetti L. (2018). Emerging functions of the EGFR in cancer. Mol. Oncol..

