# Supplementary material for: Expression of the Human Serotonin 5-HT7 Receptor Rescues Phenotype Profile and Restores Dysregulated Biomarkers in a Drosophila melanogaster Glioma Model
Source: Cells. 2022 Apr 9;11(8):1281. doi: 10.3390/cells11081281 (PMC9028361; doi:10.3390/cells11081281)
Supplement: Supplementary file 1 [file cells-11-01281-s001.zip › cells-1658825-supplementary.pdf]

Table S1. List of antibodies used in flow cytometry experiments

Antibody	Host species/ Type	Concentration used	Fluorochrome	Manufacturer/ Ref
**H3**	Rabbit/IgG	1µg/ml	Pacific Blue™	Cell signaling 12167
**Isotype H3**	Rabbit/IgG	1µg/ml	Pacific Blue™	Cell signaling 9078
**H3K4me3**	Rabbit/IgG	1µg/ml	Alexa Fluor® 647	Cell signaling 120645
**Isotype H3K4me3**	Rabbit/IgG	1µg/ml	Alexa Fluor® 647	Cell signaling 2985
**H3K4me2**	Rabbit/IgG	10µg/ml	Alexa Fluor® 647	Abcam Ab200026
**Isotype H3K4me2**	Rabbit/IgG	10µg/ml	Alexa Fluor® 647	Cell signaling 2985
**H3K9me2**	Mouse IgG	10µg/ml	Alexa Fluor® 647	Abcam Ab150115

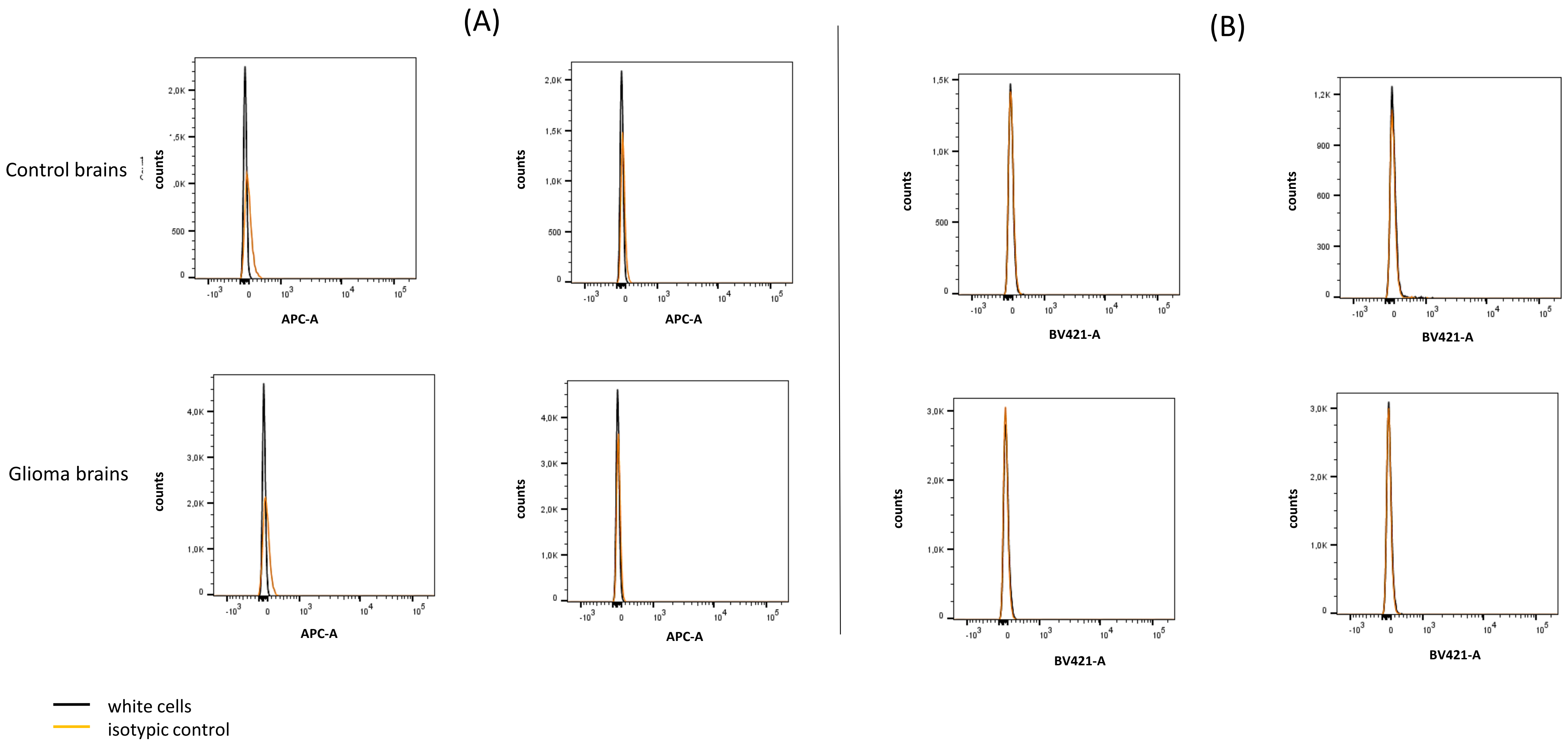

Figure S1. Flow cytometry controls. White cells and isotypic controls in gliomas and control brains:

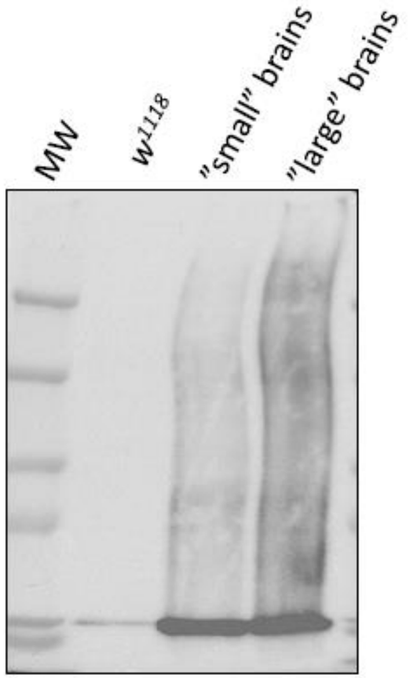

Figure S2. Expression of h5-HT7R in "small" and "large" brains expressing h5-HT7R (repo>dp110^{CAAX}; h5-HT7R; dEGFR$^{\lambda}$). Controls are w^{1118} brains. Antibodies directed against the FLAG tag were used. MW: Molecular weight marker

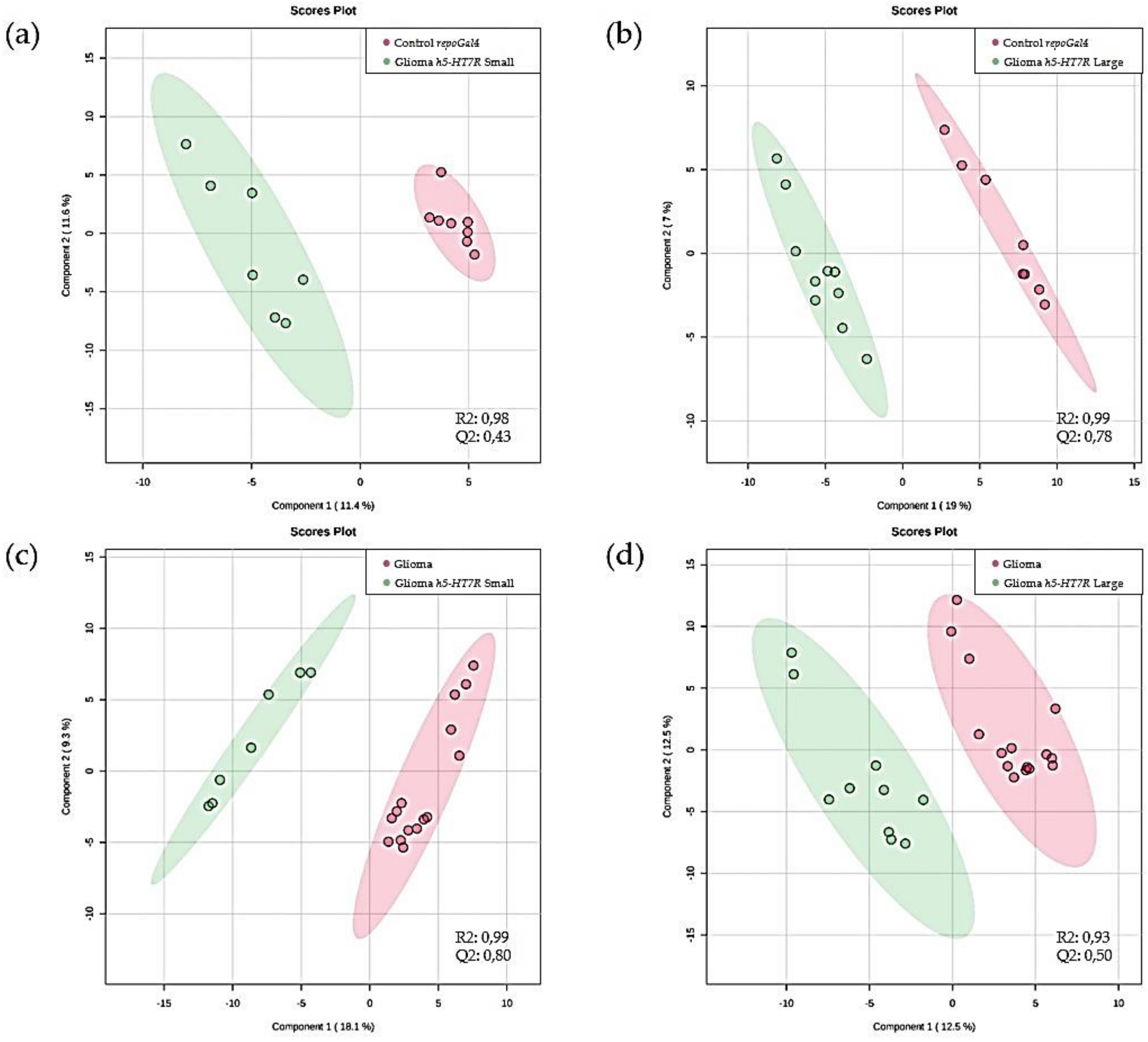

Figure S3. PLS–DA score plots. (a) Comparison of controls brains (*repo-Gal4*), and “small” brains expressing *h5-HT7R* in a glioma context, (b) Comparison of controls brains (*repo-Gal4*), and “large” brains expressing *h5-HT7R* in a glioma context. (c) Comparison of gliomas and “small” brains expressing *h5-HT7R* in a glioma context, (d) Comparison of gliomas and “large” brains expressing *h5-HT7R* in a glioma context

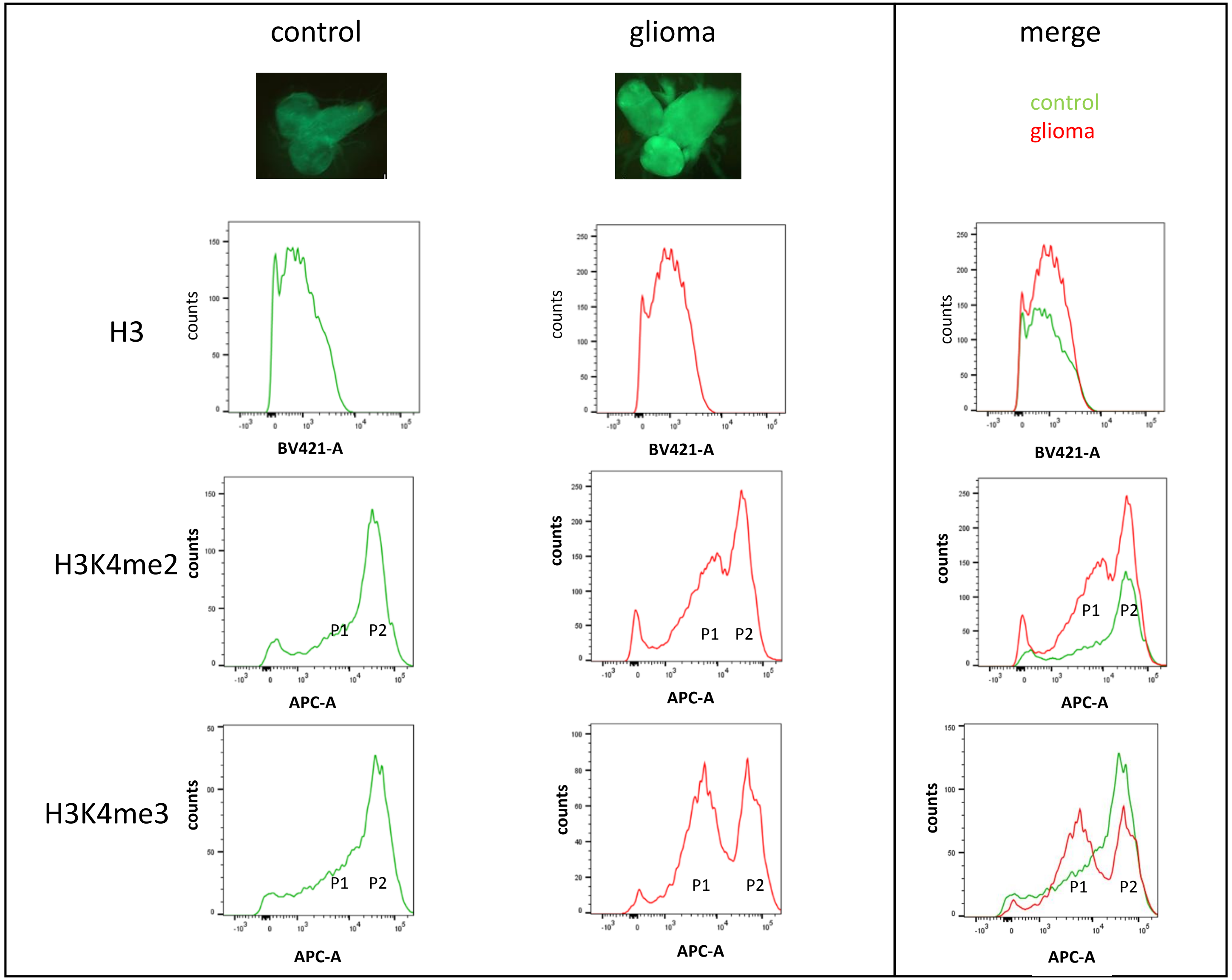

Figure S4. Analysis of H3 content and H3K4me2/3 by flow cytometry in glial cells (GFP labelled cells). Pattern of H3 labelling and H3K4me2/3 labelling in glial cells are shown for controls (*repo>GFP*) and glioma (*repo>dp110^{CAAX}; dEGFR$^{\lambda}$*). Two populations of glial cells P1 and P2 differing by their methylation rate have been identified
